# Multimodal approach to public health interventions using EGG and mobile health technologies

**DOI:** 10.3389/fpubh.2024.1520343

**Published:** 2025-01-22

**Authors:** Xiao Zhang, Han Liu, Mingyang Sun, Shuangyi Feng

**Affiliations:** ^1^School of Physical Education Institute, Yunnan Minzu University, Kunming, Yunnan, China; ^2^The Catholic University of Korea, Seoul, Republic of Korea

**Keywords:** public health interventions, PH-CLIP, EEG signal analysis, multi-scale fusion mechanism, mobile health technologies

## Abstract

**Introduction:**

Public health interventions increasingly integrate multimodal data sources, such as Electroencephalogram (EEG) data, to enhance monitoring and predictive capabilities for mental health conditions. However, traditional models often face challenges with the complexity and high dimensionality of EEG signals. While recent advancements like Contrastive Language-lmage Pre-training(CLIP) models excel in cross-modal understanding, their application to EEG-based tasks remains limited due to the unique characteristics of EEG data.

**Methods:**

In response, we introduce PH-CLIP (Public Health Contrastive Language-lmage Pretraining), a novel framework that combines CLIP's representational power with a multi-scale fusion mechanism designed specifically for EEG data within mobile health technologies. PH-CLIP employs hierarchical feature extraction to capture the temporal dynamics of EEG signals, aligning them with contextually relevant textual descriptions for improved public health insights. Through a multi-scale fusion layer, PH-CLIP enhances interpretability and robustness in EEG embeddings, thereby supporting more accurate and scalable interventions across diverse public health applications.

**Results and discussion:**

Experimental results indicate that PH-CLIP achieves significant improvements in EEG classification accuracy and mental health prediction efficiency compared to leading EEG analysis models. This framework positions PH-CLIP as a transformative tool in public health monitoring, with the potential to advance large-scale mental health interventions through integrative mobile health technologies.

## 1 Introduction

The need for robust, scalable, and interpretable systems to analyze and interpret electroencephalography (EEG) signals in public health applications has become increasingly critical. EEG provides a non-invasive, real-time monitoring approach, especially useful for mental health and neurological assessments (1). However, traditional approaches to EEG analysis are challenged by the complex, high-dimensional nature of EEG data, and the requirements for high accuracy and generalizability across diverse populations (2). PH-CLIP (Public Health Contrastive Language-Image Pretraining) with multi-scale fusion on EEG is proposed to enhance the scalability and precision of EEG interpretation by leveraging recent advances in multi-modal and multi-scale deep learning. This approach not only addresses issues of scalability but also aims to improve cross-population generalization by incorporating scalable contrastive language-image models (CLIP) adapted for EEG data, ultimately offering a powerful tool for large-scale public health monitoring and intervention (3).

To address the limitations of early symbolic AI and knowledge-based representations, traditional methods were initially used to analyze EEG data through symbolic reasoning and rule-based systems. These methods attempted to capture and encode domain knowledge, often using rule-based expert systems and symbolic models that were carefully curated by neurologists and psychologists (4). Such symbolic systems excelled in structured settings, where domain expertise could be meticulously encoded into rule sets for specific, controlled use cases (5). Despite these advantages, however, they were inherently limited in their scalability and adaptability, especially when applied to diverse EEG data in real-world public health settings. The rigid structure of symbolic systems often failed to generalize across different populations and evolving datasets, as the rules required constant refinement to handle variations in EEG signal patterns, thus constraining their utility in large-scale public health applications.

The evolution toward data-driven approaches and machine learning methods brought more flexibility and data-adaptiveness to EEG analysis. In this stage, researchers utilized traditional machine learning models such as support vector machines (SVMs) (6), k-nearest neighbors (KNN) (7), and random forest Hu et al.'s (8), applying these models to extract meaningful features from EEG signals for various health monitoring tasks. Machine learning techniques allowed for greater data-driven adaptability, as models could be trained on specific datasets and applied to classify or detect mental health states or neurological disorders (9, 10). Despite this adaptability, these approaches were limited by their reliance on feature engineering, requiring domain expertise to manually design features from raw EEG signals. Consequently, while machine learning methods provided more scalability than symbolic systems, they were still labor-intensive and often struggled with generalizability when exposed to large, heterogeneous datasets common in public health research.

The recent advances in deep learning and the development of pre-trained models like CLIP have further revolutionized the field, enabling more robust, automated feature extraction and interpretation across large, varied EEG datasets. Deep learning models, particularly convolutional neural networks (CNNs) and recurrent neural networks (RNNs), have shown significant promise in EEG interpretation by learning complex, hierarchical patterns directly from raw data. The introduction of multi-scale fusion techniques further improves these models, allowing for the combination of temporal and spatial features across multiple scales of EEG data, thereby enhancing model robustness and interpretability (11). Despite their effectiveness, these models often face issues related to scalability, particularly when extended to population-level datasets, and they require substantial computational resources (12). Pre-trained models like CLIP, designed for contrastive learning on image and text data, represent a novel opportunity to bridge this gap by enabling multi-modal fusion of EEG data with language representations, thus opening new possibilities for scalable, interpretable public health applications.

Given the limitations discussed above, PH-CLIP introduces a novel framework that combines multi-scale fusion with contrastive learning to address the scalability, adaptability, and interpretability challenges inherent in EEG analysis. Unlike traditional EEG models that often focus on single-scale features or require extensive feature engineering, PH-CLIP leverages a modified CLIP-based framework to process EEG data, enabling seamless integration of information across multiple spatial, temporal, and cross-modal scales. This multi-scale fusion allows the model to dynamically capture both localized neural patterns and broader spatiotemporal dynamics, providing a more nuanced representation of EEG signals. A key innovation of PH-CLIP lies in its ability to align EEG data with auxiliary modalities, such as contextual or textual information, through contrastive learning. This alignment not only enhances interpretability by linking neural activity to meaningful outcomes but also improves generalizability across diverse populations and datasets. These advancements make PH-CLIP uniquely suited for large-scale, real-world public health applications, where datasets are often heterogeneous and require robust adaptability. By integrating multi-scale fusion and contrastive learning into a unified framework, PH-CLIP transcends the limitations of existing EEG models, such as constrained scalability or limited interpretability. Its design ensures scalability for large datasets, adaptability to diverse populations, and accessibility for public health monitoring systems, paving the way for innovative and effective EEG-based interventions in real-world settings.

The PH-CLIP approach presents the following advantages:

- It introduces a new multi-scale fusion module, enabling simultaneous analysis of EEG data across temporal and spatial scales for improved interpretability and robustness.
- The method demonstrates high adaptability across different scenarios and populations, showing potential for efficient, scalable use in public health applications.
- Experimental results indicate significant improvements in cross-population generalization and interpretability, making it suitable for diverse public health monitoring needs.

## 2 Related work

### 2.1 Contrastive learning for EEG-based health applications

Contrastive learning has emerged as a transformative approach in the development of scalable and robust machine learning models, particularly within healthcare applications leveraging electroencephalography (EEG) data (10). The core concept of contrastive learning involves learning meaningful representations by differentiating between similar and dissimilar pairs in a given dataset, often under the CLIP (Contrastive Language-Image Pretraining) framework, which aligns multimodal data, such as text and images, in a shared embedding space. Applying CLIP to EEG data for public health monitoring involves unique challenges and adaptations, especially due to the high-dimensional, noise-sensitive, and temporally dynamic nature of EEG signals. Previous studies have shown promising results by adapting contrastive learning frameworks to capture specific health-related patterns, such as identifying biomarkers for neurological disorders, stress levels, sleep stages, or emotional states (13). The PH-CLIP model introduces scalability within the CLIP framework by incorporating multi-scale fusion techniques, which are essential for effectively capturing the multi-dimensional complexity of EEG data. This approach aligns with prior works that emphasize the necessity of multi-scale data integration, as EEG signals inherently contain features at different temporal resolutions that are relevant for various health indicators (14). The success of applying contrastive learning on EEG largely depends on effectively capturing both global and local features, and multi-scale fusion facilitates this by integrating features at different resolutions. This capability allows PH-CLIP to generalize across diverse EEG datasets, which is particularly advantageous in public health contexts where EEG-based insights might need to be adapted across varying demographic or health profiles (15). The use of contrastive learning in EEG-based health applications also requires addressing signal-specific challenges, such as artifact removal, feature extraction, and inter-subject variability. Studies have implemented several preprocessing and augmentation techniques, including Fourier transforms, wavelet decompositions, and other domain-specific transformations, to improve signal fidelity and robustness of learned representations. These methods reduce noise and ensure that the model learns from pertinent patterns, thereby enhancing its scalability across different tasks within public health. The PH-CLIP model's design considers these challenges by integrating multi-scale fusion, thereby aligning with the latest advancements in contrastive learning frameworks for healthcare applications that focus on resilience to data variability and noise while capturing meaningful health signals (16).

### 2.2 Multi-scale fusion techniques for temporal data

Multi-scale fusion techniques have gained significant traction in the context of temporal data analysis, especially where datasets exhibit features across multiple time resolutions. For EEG data, which is characterized by high temporal and frequency dynamics, multi-scale fusion serves as a powerful tool to integrate signals captured at different scales, such as short-term oscillations and long-term trends. This is critical in applications involving public health, where EEG data is analyzed to monitor conditions that manifest across varied temporal resolutions, from immediate stress responses to long-term cognitive decline. Integrating multi-scale fusion with contrastive frameworks like CLIP is particularly promising, as it allows the model to learn representations that retain coherence across these diverse temporal scales (17). In the case of PH-CLIP, the multi-scale fusion mechanism captures EEG patterns across resolutions, thus enhancing the model's capability to distinguish between meaningful signals and background noise. This technique is achieved by first transforming EEG data into representations at various scales, often through down-sampling or spectral filtering techniques, followed by a hierarchical fusion process that aggregates features into a cohesive representation. Prior studies in other fields, such as speech recognition and activity monitoring, have shown that multi-scale fusion enhances robustness and feature richness by aligning information from short and long time spans. For EEG applications, this method allows for an adaptable model structure that accommodates both immediate and cumulative health indicators (18). Beyond EEG, multi-scale fusion methods have been explored in other temporal data domains, indicating their broad applicability and utility. Methods such as convolutional and recurrent neural networks, combined with attention mechanisms, have been shown to improve multi-scale processing in fields where complex temporal dependencies exist. In EEG data analysis for public health, these methods enable a more nuanced understanding of brain activity, supporting predictive modeling of health outcomes (19). PH-CLIP leverages these techniques to address the inherent complexity of EEG signals and aims to improve generalization across diverse health contexts. The integration of multi-scale fusion within PH-CLIP establishes a scalable model architecture that can potentially support applications from individual health monitoring to broader epidemiological studies (20).

### 2.3 Applications of CLIP in public health monitoring

Contrastive learning frameworks, particularly the CLIP model, have shown substantial potential for enhancing public health monitoring by enabling scalable, cross-modal representations of health-related data. The CLIP model's original design aligns textual and visual information, but adaptations for EEG data can allow the alignment of EEG signals with other health-related data types, such as clinical annotations or demographic information. By employing the PH-CLIP model in this context, researchers and public health professionals could leverage a unified model that draws on rich EEG signals to produce health insights relevant to diverse monitoring needs, including early detection of mental health conditions, stress assessment, or cognitive state analysis (21). Adaptations of CLIP for public health applications have been explored by aligning biomedical signals with complementary data modalities to facilitate comprehensive health assessments. In EEG analysis, this approach can capture associations between brain activity patterns and reported health outcomes, thus enabling a cross-modal understanding of health conditions. The application of such a model in public health has implications for large-scale, non-invasive monitoring systems that could be deployed in community or clinical settings to monitor mental health trends or the impact of environmental stressors on neurological health. PH-CLIP's design, focusing on scalability and multi-scale fusion, enhances this applicability by allowing robust EEG representation across varied health contexts, making it particularly suited for generalizable public health insights (22). Previous research on public health applications of CLIP has also focused on the challenge of model interpretability, especially given the need for transparent models in health contexts. Adaptations such as explainable AI (XAI) techniques can be integrated with PH-CLIP to interpret which EEG features contribute to specific health predictions, thereby supporting actionable insights for public health interventions. This interpretability is critical in understanding the specific neurological markers that may indicate cognitive decline, emotional stress, or other health states relevant to public health. Overall, PH-CLIP's scalability and interpretability make it a promising approach for deploying CLIP models in public health settings, supporting large-scale and accessible health monitoring systems that adapt to the diverse needs of a population (23).

### 2.4 Advances in multi-modal EEG data integration

Recent years have witnessed significant progress in EEG data analysis, particularly in the integration of multi-modal data to enhance robustness, interpretability, and generalizability. Traditional EEG analysis methods primarily rely on single-modal feature extraction, focusing on temporal or spectral characteristics of neural signals. While these approaches have achieved notable success in applications such as mental health monitoring and cognitive state recognition, they often fail to capture the contextual and environmental factors influencing EEG signals, limiting their applicability in real-world scenarios. One promising direction involves the integration of auxiliary data modalities, such as textual, visual, or physiological signals, to complement EEG analysis. For example, Gaidai and Yihan (24) proposed a fusion framework combining EEG and eye-tracking data for improved emotion recognition, demonstrating that cross-modal feature alignment enhances model performance in complex tasks. Similarly, Han et al. (25) utilized speech and EEG data jointly to analyze cognitive workload, highlighting the potential of multi-modal integration to capture subtle interactions between brain activity and external stimuli. Deep learning methods, particularly those leveraging attention mechanisms, have played a pivotal role in advancing multi-modal EEG integration. Transformer-based architectures, as explored in Gaidai et al. (26), align EEG features with video data to understand affective states in dynamic environments. However, these models often face challenges related to computational efficiency and overfitting in small datasets. Graph Neural Networks (GNNs), such as those used in Qeadan et al. (27), have also shown promise by modeling spatial dependencies between EEG electrodes and correlating them with auxiliary data sources, such as motion or heart rate. Despite these advances, existing models often struggle with scalability and generalizability across diverse populations and datasets. Many approaches require extensive pre-processing or domain-specific feature engineering, which hinders their deployment in real-world applications. Moreover, multi-modal alignment techniques often lack interpretability, making it difficult to derive actionable insights from the integrated features. To address these limitations, PH-CLIP leverages a contrastive learning framework modified for EEG data, enabling robust alignment of EEG features with auxiliary modalities such as textual or contextual information. By integrating multi-scale fusion with cross-modal learning, PH-CLIP captures the complex interactions between neural activity and external factors, ensuring adaptability and scalability for large-scale public health applications. This positions PH-CLIP as a significant advancement over existing methods, offering a unified framework for EEG-based multi-modal analysis.

## 3 Method

### 3.1 Overview

**Figure 1:**
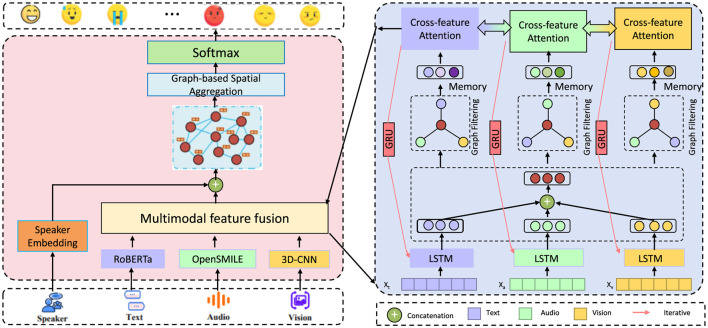
Diagram of HealthNet, an advanced multi-modal forecasting framework for public health prediction, integrating neural architectures like RNNs, transformers, and graph-based layers. The model processes data from multiple modalities—text, audio, and visual—via specialized feature fusion and cross-feature attention mechanisms to capture complex epidemiological and social interactions. Designed for flexibility, HealthNet incorporates attention layers to prioritize key features and applies bias mitigation techniques to address socioeconomic and demographic disparities, ensuring accurate, interpretable public health forecasts.

In this section, we present an innovative forecasting framework specifically designed to address challenges in public health prediction. The framework leverages multi-modal data sources, advanced neural architectures, and specialized strategies for bias mitigation and model interpretability. The approach is structured into several key modules, each contributing a specialized component to ensure accurate, resilient, and meaningful forecasting in the context of public health. In the subsequent sections, we first detail the Mathematical Formulation of our forecasting problem. This includes a comprehensive definition of public health parameters, such as epidemiological variables, social determinants, and health system factors, which are represented within a high-dimensional, multi-modal data space. We introduce notation to formalize the relationships between input features, health outcomes, and temporal dependencies, ensuring the setup for a robust predictive modeling approach. Following this foundational setup, we introduce the Proposed Model Architecture. Our model, denoted as HealthNet, combines features from recurrent neural networks (RNNs), transformers, and graph-based layers to capture complex interactions between epidemiological, social, and behavioral variables. The model design is optimized for flexibility across various public health datasets and incorporates mechanisms to handle both structured and unstructured data sources. HealthNet integrates attention mechanisms to prioritize critical features dynamically, allowing for nuanced handling of time-series data in public health applications (as shown in Figure 1).

We then elaborate on the Prediction Enhancement Strategies, which further bolster our model's capabilities in real-world scenarios. These strategies include domain-specific adjustments, such as debiasing methods to mitigate the impact of socioeconomic and demographic disparities that can skew health outcomes. Additionally, we describe a fine-tuning procedure tailored for adapting the model to varying levels of data sparsity and noise, a common challenge in public health data. Finally, we provide a comprehensive summary of Implementation and Optimization Techniques applied to our model. These techniques encompass parameter tuning, model validation protocols, and specific metrics used to evaluate forecast accuracy in public health contexts. We also discuss interpretability strategies employed to make HealthNet's predictions accessible and actionable for public health professionals and policymakers. Through this structured approach, the proposed framework offers a comprehensive solution for public health forecasting, designed to adapt to diverse datasets and address real-world forecasting challenges effectively.

### 3.2 Preliminaries

In this section, we formalize the public health forecasting problem and establish the mathematical notation and structures necessary for the proposed framework. Our objective is to predict specific health outcomes, such as disease incidence, hospitalization rates, or mortality, based on a set of epidemiological, social, and environmental indicators. The challenge involves capturing complex dependencies across time and between different indicators while addressing issues related to data sparsity, heterogeneity, and potential biases inherent in public health data.

Let $X={\left\{{\text{x}}_{t}\right\}}_{t=1}^{T}$ denote a sequence of multi-dimensional input vectors, where ${\text{x}}_{t}=\left\{x_{t}^{\left(1\right)},x_{t}^{\left(2\right)},\ldots ,x_{t}^{\left(M\right)}\right\}$ represents the vector of M features observed at time *t*. Each feature $x_{t}^{\left(m\right)}$ captures information relevant to public health, such as the number of reported disease cases, environmental factors, or sociodemographic characteristics. For each time step *t*, we aim to predict a health outcome variable *y*_*t*_, which could represent disease incidence, hospitalization rates, or other health indicators of interest.

The public health forecasting task can be framed as finding a function *f* that maps the historical data X to the predicted outcomes $Y={\left\{y_{t}\right\}}_{t=1}^{T}$ over the observed time horizon *T*. Mathematically, we seek:


(1) $$\begin{array}{l} {ŷ}_{t}=f\left({\text{x}}_{t},{\text{x}}_{t-1},\ldots ,{\text{x}}_{t-L}\right), \end{array}$$

where *L* is the window size for historical data considered in each prediction. The function *f* is designed to capture temporal dependencies and correlations between different input features, allowing the model to adapt to dynamic changes in public health conditions.

Public health outcomes often exhibit temporal autocorrelation, where past values significantly influence future predictions. We model these dependencies through sequential inputs **x**_*t*−*L*:*t*_, where **x**_*t*−*L*:*t*_ denotes the concatenated vectors {**x**_*t*_, **x**_*t*−1_, …, **x**_*t*−*L*_}, capturing the recent history of feature observations.

Let ${\text{X}}^{\left(m\right)}={\left\{x_{t}^{\left(m\right)}\right\}}_{t=1}^{T}$ represent the time series for feature *m* over the entire time horizon. We hypothesize that specific interactions between these features, such as the influence of environmental conditions on disease spread, play a crucial role in accurate forecasting. We therefore define cross-feature dependency functions ϕ_*m*_, such that:


(2) $$\begin{array}{l} {\text{z}}_{t}^{\left(m\right)}=\phi_{m}\left({\text{x}}_{t}^{\left(1\right)},{\text{x}}_{t}^{\left(2\right)},\ldots ,{\text{x}}_{t}^{\left(M\right)}\right), \end{array}$$

where ${\text{z}}_{t}^{\left(m\right)}$ denotes a transformed representation of feature *m* that incorporates information from other features at time *t*.

To allow the model to adaptively focus on the most relevant time points and features, we introduce an attention mechanism $\alpha_{t,\tau }^{\left(m\right)}$, where τ denotes a lagged time step relative to *t*. The attention weights $\alpha_{t,\tau }^{\left(m\right)}$ satisfy $\overset{L}{\underset{\tau =0}{\sum }}\alpha_{t,\tau }^{\left(m\right)}=1$, and are computed as:


(3) $$\begin{array}{l} \alpha_{t,\tau }^{\left(m\right)}=\frac{\exp\left(e_{t,\tau }^{\left(m\right)}\right)}{\overset{L}{\underset{\tau^{\prime }=0}{\sum }}\exp\left(e_{t,\tau^{\prime }}^{\left(m\right)}\right)}, \end{array}$$

where $e_{t,\tau }^{\left(m\right)}$ is a score function that evaluates the relevance of the past time point *t* − τ for predicting *y*_*t*_ based on feature *m*. The output of the attention mechanism, ${\tilde{\text{x}}}_{t}^{\left(m\right)}$, is computed as:


(4) $$\begin{array}{l} {\tilde{\text{x}}}_{t}^{\left(m\right)}=\overset{L}{\underset{\tau =0}{\sum }}\alpha_{t,\tau }^{\left(m\right)}{\text{x}}_{t-\tau }^{\left(m\right)}. \end{array}$$

Given the spatial dependencies often observed in public health data, we define an undirected graph *G* = (*V, E*), where *V* represents locations and *E* denotes edges representing relationships. Each node *v* ∈ *V* is associated with a vector **x**_*v*_ of health indicators specific to that location. The influence of neighboring locations u∈N(v) on location *v* at time *t* is captured as:


(5) $$\begin{array}{l} {\text{h}}_{v,t}=\underset{u\in N\left(v\right)}{\sum }w_{uv}{\text{x}}_{u,t}, \end{array}$$

where *w*_*uv*_ is a weight capturing the strength of the interaction between nodes *u* and *v*. This graph structure allows us to model region-specific factors and their impacts on local health outcomes, capturing patterns of spatial dependency.

To optimize the forecasting accuracy, we define a prediction loss $L\left({ŷ}_{t},y_{t}\right)$ that measures the error between the predicted and actual outcomes. A common choice is mean squared error (MSE):


(6) $$\begin{array}{l} L\left({ŷ}_{t},y_{t}\right)=\frac{1}{T}\overset{T}{\underset{t=1}{\sum }}{\left({ŷ}_{t}-y_{t}\right)}^{2}. \end{array}$$

This loss is minimized over the training set to tune the parameters of the function *f*, ensuring that the model learns accurate temporal and feature-based dependencies for public health forecasting.

### 3.3 HealthNet model architecture

Our proposed model, termed HealthNet, is designed to capture the intricate temporal, spatial, and cross-feature relationships present in public health data. HealthNet leverages a hybrid architecture combining recurrent neural networks (RNNs), transformer-based attention layers, and graph convolutional networks (GCNs) to account for temporal dependencies, contextual relevance, and spatial correlations, respectively. This section provides a detailed description of HealthNet's components and their integration to achieve accurate and interpretable forecasting in public health settings. The HealthNet model consists of three main modules: Temporal Encoding, Cross-feature Attention Layer, and Graph-based Spatial Aggregation.

**Temporal encoding**

**Figure 2:**
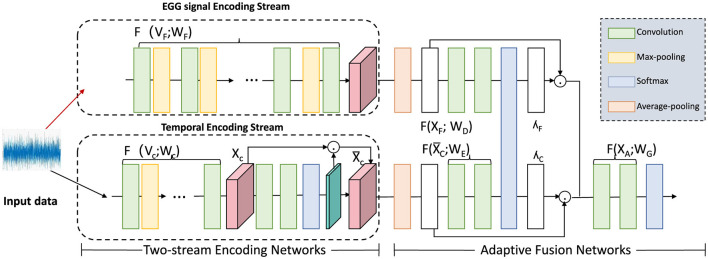
Diagram illustrating a two-stream encoding network for temporal data processing. The framework comprises an EGG signal encoding stream and a temporal encoding stream, each utilizing convolution, max-pooling, and softmax operations to process input signals. These streams feed into an adaptive fusion network that applies multiple transformations, including average-pooling, to integrate information across the channels. This architecture captures both signal-specific and temporal dependencies, facilitating precise, and adaptive temporal encoding for downstream analysis.

In our temporal encoding framework, we utilize a multi-layered recurrent neural network (RNN) model to capture the complex sequential dependencies within public health data, where historical observations often impact future outcomes significantly. Each temporal step contains multiple features, and the RNN encodes these into hidden states over time. Let ${\text{z}}_{t}^{\left(k\right)}$ denote the hidden state at time *t* for feature *k*. This state evolves recursively based on the previous hidden state ${\text{z}}_{t-1}^{\left(k\right)}$ and the current input ${\text{y}}_{t}^{\left(k\right)}$, as formulated:


(7) $$\begin{array}{l} {\text{z}}_{t}^{\left(k\right)}=\text{RNN}\left({\text{y}}_{t}^{\left(k\right)},{\text{z}}_{t-1}^{\left(k\right)};\gamma_{\text{RNN}}\right), \end{array}$$

where γ_RNN_ represents the learned parameters of the RNN model. The hidden state ${\text{z}}_{t}^{\left(k\right)}$ captures the temporal dependencies for feature *k* across past intervals, progressively updating to incorporate new information with each time step. By stacking multiple RNN layers, the model can aggregate high-level temporal dependencies, enhancing its capacity to understand long-range patterns (as shown in Figure 2).

To further optimize feature relevance dynamically, a temporal attention mechanism is introduced, assigning importance scores $\beta_{t}^{\left(k\right)}$ to the hidden states based on their significance for the prediction target. The attention score calculation integrates an alignment mechanism over hidden states, represented by:


(8) $$\begin{array}{l} \beta_{t}^{\left(k\right)}=\frac{\exp\left({\text{q}}^{⊤}\cdot \text{tanh}\left({\text{Q}}_{z}{\text{z}}_{t}^{\left(k\right)}+{\text{c}}_{z}\right)\right)}{\underset{t^{\prime }}{\sum }\exp\left({\text{q}}^{⊤}\cdot \text{tanh}\left({\text{Q}}_{z}{\text{z}}_{t^{\prime }}^{\left(k\right)}+{\text{c}}_{z}\right)\right)}, \end{array}$$

where **Q**_*z*_ and **c**_*z*_ are learnable matrices and biases, and **q** is a weight vector that projects hidden states into an attention-relevant domain. This mechanism ensures that more informative hidden states contribute proportionally to the model's temporal context.

The temporal context vector, **s**_*t*_, is generated by combining the hidden states with their respective attention scores, effectively summarizing relevant temporal information as:


(9) $$\begin{array}{l} {\text{s}}_{t}=\underset{k}{\sum }\beta_{t}^{\left(k\right)}{\text{z}}_{t}^{\left(k\right)}, \end{array}$$

where **s**_*t*_ is then used as input to the prediction layer. This context vector adapts dynamically to the data at each time step, allowing the model to focus on the most critical temporal signals.

To capture both short-term and long-term dependencies effectively, we add a gating mechanism in the RNN hidden state computation. Let ${\text{g}}_{t}^{\left(k\right)}$ denote a forget gate and ${\text{i}}_{t}^{\left(k\right)}$ an input gate, controlling the memory retention and update dynamics:


(10) $$\begin{array}{l} {\text{g}}_{t}^{\left(k\right)}=\sigma \left({\text{W}}_{g}{\text{y}}_{t}^{\left(k\right)}+{\text{U}}_{g}{\text{z}}_{t-1}^{\left(k\right)}+{\text{b}}_{g}\right), \end{array}$$

(11) $$\begin{array}{l} {\text{i}}_{t}^{\left(k\right)}=\sigma \left({\text{W}}_{i}{\text{y}}_{t}^{\left(k\right)}+{\text{U}}_{i}{\text{z}}_{t-1}^{\left(k\right)}+{\text{b}}_{i}\right), \end{array}$$

where σ is the sigmoid activation, and **W**_*g*_, **W**_*i*_, **U**_*g*_, **U**_*i*_, **b**_*g*_, **b**_*i*_ are learnable parameters. The updated hidden state then integrates both the gated prior state and new input information:


(12) $$\begin{array}{l} {\text{z}}_{t}^{\left(k\right)}={\text{g}}_{t}^{\left(k\right)}⊙{\text{z}}_{t-1}^{\left(k\right)}+{\text{i}}_{t}^{\left(k\right)}⊙\text{tanh}\left({\text{W}}_{z}{\text{y}}_{t}^{\left(k\right)}+{\text{U}}_{z}{\text{z}}_{t-1}^{\left(k\right)}+{\text{b}}_{z}\right), \end{array}$$

where ⊙ denotes element-wise multiplication, ensuring that each hidden state incorporates both immediate and historical data. This refined temporal encoding approach enables more robust and context-sensitive predictions.

**Cross-feature attention layer**

HealthNet incorporates a specialized cross-feature attention layer that dynamically models interdependencies among public health features, allowing the network to account for complex interactions in health-related data. Recognizing that certain features may amplify or attenuate the impact of others, this attention mechanism enhances the model's ability to adjust its focus across features in response to changing conditions over time. By attending to other features, each feature representation can adaptively prioritize influential relationships, improving predictive accuracy.

Let the set of feature embeddings at time *t* be denoted by $\left\{{\text{h}}_{t}^{\left(1\right)},{\text{h}}_{t}^{\left(2\right)},\ldots ,{\text{h}}_{t}^{\left(M\right)}\right\}$, where each ${\text{h}}_{t}^{\left(m\right)}$ represents the embedding for feature *m*. The cross-feature attention mechanism calculates a set of attention weights $\beta_{t}^{\left(m,m^{\prime }\right)}$, which assign varying levels of influence from feature *m*′ to feature *m* based on their current contextual relevance. These weights are computed as:


(13) $$\begin{array}{l} \beta_{t}^{\left(m,m^{\prime }\right)}=\frac{\exp\left(\frac{{\text{h}}_{t}^{\left(m\right)}\cdot {\text{h}}_{t}^{\left(m^{\prime }\right)}}{\tau }\right)}{\underset{m^{\prime \prime }}{\sum }\exp\left(\frac{{\text{h}}_{t}^{\left(m\right)}\cdot {\text{h}}_{t}^{\left(m^{\prime \prime }\right)}}{\tau }\right)}, \end{array}$$

where:

- $\beta_{t}^{\left(m,m^{\prime }\right)}$ represents the attention weight quantifying the influence of feature *m*′ on feature *m* at time *t*.
- ${\text{h}}_{t}^{\left(m\right)}$ and ${\text{h}}_{t}^{\left(m^{\prime }\right)}$ are the embeddings of features *m* and *m*′ at time *t*, respectively.
· denotes the dot product, which measures the similarity between feature embeddings.
- τ > 0 is the temperature parameter that controls the sharpness of the attention distribution:
  ∘ Smaller τ values concentrate attention on a few features by amplifying the relative differences in similarity.
  ∘ Larger τ values distribute attention more evenly across features by reducing sensitivity to similarity differences.

Once the attention scores $\beta_{t}^{\left(m,m^{\prime }\right)}$ are obtained, a cross-feature context vector ${\text{g}}_{t}^{\left(m\right)}$ is generated for each feature *m* by summing the influence-weighted embeddings of all other features:


(14) $$\begin{array}{l} {\text{g}}_{t}^{\left(m\right)}=\underset{m^{\prime }}{\sum }\beta_{t}^{\left(m,m^{\prime }\right)}{\text{h}}_{t}^{\left(m^{\prime }\right)}. \end{array}$$

This context vector ${\text{g}}_{t}^{\left(m\right)}$ captures the aggregated influence of the other features on feature *m* at the current time step. By integrating ${\text{g}}_{t}^{\left(m\right)}$ with the original embedding ${\text{h}}_{t}^{\left(m\right)}$, we obtain an enhanced feature representation that reflects both the inherent properties of *m* and the dynamically computed influence from other features. This integration is formalized as follows:


(15) $$\begin{array}{l} {\tilde{\text{h}}}_{t}^{\left(m\right)}=\lambda {\text{h}}_{t}^{\left(m\right)}+\left(1-\lambda \right){\text{g}}_{t}^{\left(m\right)}, \end{array}$$

where λ is a learnable parameter that adjusts the balance between the original feature embedding ${\text{h}}_{t}^{\left(m\right)}$ and the cross-feature context ${\text{g}}_{t}^{\left(m\right)}$. This weighted sum allows the model to dynamically modulate the influence of cross-feature information according to the context, providing flexibility to focus on relevant interactions without overriding feature-specific details.

Furthermore, an additional self-attention layer can be applied to the enhanced representations ${\tilde{\text{h}}}_{t}^{\left(m\right)}$ to refine the model's focus across features further. This secondary attention mechanism computes an updated representation for each feature that incorporates second-order interdependencies:


(16) $$\begin{array}{l} \alpha_{t}^{\left(m\right)}=\frac{\exp\left({\text{w}}^{⊤}\cdot \text{tanh}\left({\text{W}}_{h}{\tilde{\text{h}}}_{t}^{\left(m\right)}+{\text{b}}_{h}\right)\right)}{\underset{m^{\prime }}{\sum }\exp\left({\text{w}}^{⊤}\cdot \text{tanh}\left({\text{W}}_{h}{\tilde{\text{h}}}_{t}^{\left(m^{\prime }\right)}+{\text{b}}_{h}\right)\right)}, \end{array}$$

where **W**_*h*_, **b**_*h*_, and **w** are learnable parameters that govern the attention distribution across enhanced features. The resulting representation leverages the dynamic inter-feature relationships captured by the cross-feature attention, contributing to more context-aware predictions that reflect both temporal dependencies and feature interactions.

**Graph-based spatial aggregation**

Public health outcomes often exhibit strong spatial correlations influenced by factors such as geographic proximity, population mobility, and shared environmental conditions. For example, infectious diseases may spread across neighboring regions, while socioeconomic factors can lead to similar health outcomes in proximate areas. To effectively capture these spatial dependencies, HealthNet employs a Graph Convolutional Network (GCN) layer that enables information exchange across interconnected locations. The spatial relationships between regions are encoded in a predefined adjacency matrix **A**, where each node represents a geographic area, and edges capture spatial proximity, travel patterns, or other linking factors.

For each geographic location *v*, the GCN layer aggregates information from its neighboring regions u∈N(v) based on the adjacency structure. This aggregation produces a spatial feature vector **s**_*v,t*_ at time *t*, which integrates information from nearby locations into a unified representation. The aggregation process is formulated as:


(17) $$\begin{array}{l} {\text{s}}_{v,t}=\sigma \left(\underset{u\in N\left(v\right)}{\sum }\frac{{\text{A}}_{uv}}{\sqrt{{\text{D}}_{vv}{\text{D}}_{uu}}}{\text{W}}_{g}{\text{h}}_{u,t}+{\text{b}}_{g}\right), \end{array}$$

where **h**_*u,t*_ represents the feature embedding of neighboring location *u* at time *t*, **A**_*uv*_ specifies the connection strength between locations *u* and *v* in the adjacency matrix, and **D**_*vv*_ and **D**_*uu*_ are degree matrix entries used to normalize the aggregation. The parameters **W**_*g*_ and **b**_*g*_ are learnable weights of the GCN, and σ(·) is a non-linear activation function such as ReLU or sigmoid. The normalized adjacency structure ensures stability and accounts for varying connectivity across regions.

To capture higher-order spatial dependencies, a multi-hop mechanism extends the aggregation process to include information from more distant regions. For a given location *v*, the spatial feature vector at hop *k*, denoted as ${\text{s}}_{v,t}^{\left(k\right)}$, is recursively defined as:


(18) $$\begin{array}{l} {\text{s}}_{v,t}^{\left(k\right)}=\sigma \left(\underset{u\in N\left(v\right)}{\sum }\frac{{\text{A}}_{uv}}{\sqrt{{\text{D}}_{vv}{\text{D}}_{uu}}}{\text{W}}_{g}^{\left(k\right)}{\text{s}}_{u,t}^{\left(k-1\right)}+{\text{b}}_{g}^{\left(k\right)}\right), \end{array}$$

where ${\text{s}}_{v,t}^{\left(0\right)}={\text{h}}_{v,t}$ is the initial feature embedding for location *v*, and ${\text{W}}_{g}^{\left(k\right)}$ and ${\text{b}}_{g}^{\left(k\right)}$ are learnable parameters for the *k*-th hop. This recursive process enables the model to aggregate information from both local and distant regions, incorporating broader spatial contexts.

Multi-hop features are combined through a weighted sum to produce the final spatial representation:


(19) $$\begin{array}{l} {\text{s}}_{v,t}=\overset{K}{\underset{k=0}{\sum }}\alpha^{\left(k\right)}{\text{s}}_{v,t}^{\left(k\right)}, \end{array}$$

where α^(*k*)^ are learnable weights that determine the relative importance of each hop. This adaptive weighting mechanism allows the model to balance local and global influences, depending on the spatial structure and the specific task requirements.

**Prediction layer and loss function**

The final prediction ŷ_*t*_ is generated by combining the outputs from the temporal encoding, cross-feature attention, and spatial aggregation layers, each of which contributes unique information to the overall prediction. Specifically, the temporal context vector **c**_*t*_ encapsulates temporal dependencies, the cross-feature attention vector **g**_*t*_ models inter-feature relationships, and the spatial aggregation vector **s**_*t*_ captures spatial correlations across locations. By concatenating these vectors, the prediction layer can access a rich, multi-dimensional representation of the data at time *t*. Formally, the prediction ŷ_*t*_ is computed as:


(20) $$\begin{array}{l} {ŷ}_{t}={\text{w}}_{p}^{⊤}\left[{\text{c}}_{t};{\text{g}}_{t};{\text{s}}_{t}\right]+b_{p}, \end{array}$$

where:

- ŷ_*t*_ represents the predicted health outcome at time *t*.
- **c**_*t*_ is the temporal context vector, capturing sequential dependencies and patterns from historical data.
- **g**_*t*_ refers to the cross-feature context vector, modeling interdependencies among features and dynamically adapting based on their relationships.
- **s**_*t*_ represents the spatial aggregation vector, capturing spatial correlations and influences from neighboring regions.
- [**c**_*t*_; **g**_*t*_; **s**_*t*_] denotes the concatenation of the vectors, integrating temporal, cross-feature, and spatial information into a unified representation.
- **w**_*p*_ is a learnable weight vector in the prediction layer, responsible for mapping the combined features to the predicted outcome.
- *b*_*p*_ is a learnable scalar bias term that adjusts the prediction for better fitting.

To train HealthNet, we aim to minimize the discrepancy between the predicted values ŷ_*t*_ and the true values *y*_*t*_ by optimizing the parameters across the entire network. This is accomplished through a mean squared error (MSE) loss function, defined as:


(21) $$\begin{array}{l} L=\frac{1}{T}\overset{T}{\underset{t=1}{\sum }}{\left({ŷ}_{t}-y_{t}\right)}^{2}, \end{array}$$

where *T* is the total number of time steps, and *y*_*t*_ represents the ground truth health outcome at time *t*. The MSE loss penalizes large deviations between predictions and actual values, driving the model to learn accurate patterns in temporal, spatial, and feature-based dependencies.

To enhance model training, we also introduce regularization terms to the loss function, addressing potential issues with overfitting due to the complex, high-dimensional input space. The augmented loss function is expressed as:


(22) $$\begin{array}{l} L=\frac{1}{T}\overset{T}{\underset{t=1}{\sum }}{\left({ŷ}_{t}-y_{t}\right)}^{2}+\lambda_{1}\|{\text{w}}_{p}|{|}^{2}+\lambda_{2}\|{\text{W}}_{g}|{|}^{2}+\lambda_{3}\|{\text{W}}_{h}|{|}^{2}, \end{array}$$

where λ_1_, λ_2_, and λ_3_ are regularization coefficients for the prediction weights **w**_*p*_, spatial aggregation weights **W**_*g*_, and cross-feature attention weights **W**_*h*_, respectively. These regularization terms mitigate the risk of overfitting by constraining the magnitude of the weights, promoting a more generalized and stable model.

In addition to MSE, we employ a temporal consistency regularization that encourages stability in predictions across consecutive time steps. This additional regularization term $L_{\text{temp}}$ is defined as:


(23) $$\begin{array}{l} L_{\text{temp}}=\frac{1}{T-1}\overset{T}{\underset{t=2}{\sum }}{\left({ŷ}_{t}-{ŷ}_{t-1}\right)}^{2}, \end{array}$$

where $L_{\text{temp}}$ penalizes abrupt changes in predicted values across time steps, particularly useful in health forecasting where outcomes typically evolve gradually. The final loss function combining MSE, weight regularization, and temporal consistency is:


(24) $$\begin{array}{l} L_{\text{total}}=L+\alpha L_{\text{temp}}, \end{array}$$

where α is a balancing coefficient that controls the influence of the temporal consistency term. This comprehensive loss formulation enables HealthNet to learn from multi-dimensional dependencies while preserving temporal smoothness, leading to more robust and reliable health outcome forecasts.

**Multi-scale fusion process**

The multi-scale fusion mechanism integrates features across both spatial and temporal dimensions to enhance robustness and interpretability. Temporal features are extracted at multiple resolutions by applying down-sampling and spectral filtering to the raw temporal data, capturing short-term and long-term patterns. A temporal attention mechanism is introduced to assign weights to each resolution dynamically, where the weight for temporal resolution *t* is calculated as:


(25) $$\begin{array}{l} \alpha^{\left(t\right)}=\frac{\exp\left(e^{\left(t\right)}\right)}{\underset{t^{\prime }}{\sum }\exp\left(e^{\left(t^{\prime }\right)}\right)}, \end{array}$$

and *e*^(*t*)^ is a learnable scoring function. The aggregated temporal features are computed as:


(26) $$\begin{array}{l} X_{\text{temporal}}=\underset{t}{\sum }\alpha^{\left(t\right)}X^{\left(t\right)}. \end{array}$$

Spatial features are processed using a Graph Convolutional Network (GCN), where each node corresponds to a spatial location and edges represent spatial relationships. The spatial feature at node *v* for layer *k* is given by:


(27) $$\begin{array}{l} h_{v}^{\left(k\right)}=\sigma \left(\underset{u\in N\left(v\right)}{\sum }{Ã}_{vu}W^{\left(k\right)}h_{u}+b^{\left(k\right)}\right), \end{array}$$

where Ã_*vu*_ is the normalized adjacency matrix, *W*^(*k*)^ is a learnable weight matrix, and σ is a non-linear activation function. To capture higher-order dependencies, multi-hop GCN layers are applied, enabling interactions across multiple spatial hops:


(28) $$\begin{array}{l} s_{v}^{\left(k\right)}=\sigma \left(\underset{u\in N\left(v\right)}{\sum }\frac{A_{vu}}{\sqrt{D_{vv}D_{uu}}}W^{\left(k\right)}s_{u}^{\left(k-1\right)}+b^{\left(k\right)}\right). \end{array}$$

The spatial and temporal representations are combined through an adaptive fusion mechanism, which dynamically adjusts the contributions of each using a learnable parameter λ:


(29) $$\begin{array}{l} X_{\text{fused}}=\lambda X_{\text{temporal}}+\left(1-\lambda \right)X_{\text{spatial}}. \end{array}$$

To further refine the representation, a hierarchical attention mechanism is applied to prioritize specific scales and regions, producing the final integrated feature representation:


(30) $$\begin{array}{l} X_{\text{final}}=\text{Attention}\left(X_{\text{fused}},\text{context}\right). \end{array}$$

This comprehensive multi-scale fusion process ensures that critical temporal and spatial patterns are effectively captured and integrated, providing a robust representation for downstream tasks.

### 3.4 Adaptive contextual adjustment mechanism

In real-world public health forecasting, data quality and availability can vary significantly across different time periods and regions, affecting model reliability. To address these challenges, we propose an Adaptive Contextual Adjustment Mechanism (ACAM) within HealthNet. ACAM is designed to dynamically adjust predictions based on the contextual uncertainty and variability of input features. This mechanism ensures that HealthNet's predictions remain robust even under conditions of sparse, noisy, or incomplete data.

**Confidence weighting for feature reliability**

**Figure 3:**
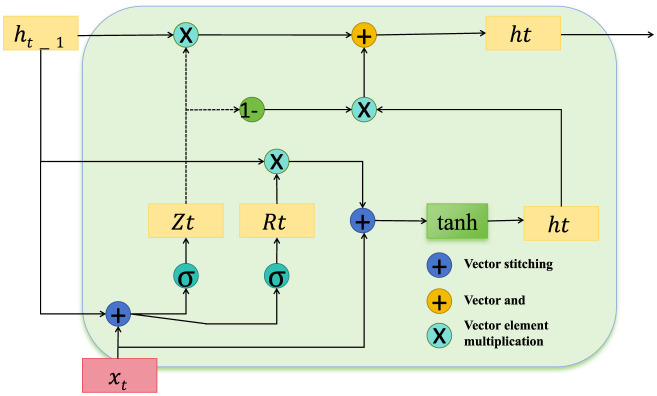
Diagram depicting the confidence-weighting mechanism within the temporal encoding module, designed to enhance feature reliability in public health data. The structure includes key operations for vector stitching, element-wise multiplication, and gating, with components *Z*_*t*_ and *R*_*t*_ modulating feature influence based on their stability. This architecture ensures adaptive feature weighting, prioritizing reliable features, and dynamically adjusting confidence scores to mitigate the impact of noisy data on downstream processing.

In the domain of public health data analysis, certain features consistently demonstrate higher predictive stability, while others may exhibit variability due to external influences or measurement inconsistencies. To address this variability, ACAM incorporates a confidence weighting module that assigns a dynamic reliability score $\gamma_{t}^{\left(m\right)}$ to each feature *m* at time *t*, modulating the influence of each feature according to its reliability. This approach enables the model to prioritize features with stable predictive power, enhancing robustness against noisy or inconsistent data (as shown in Figure 3).

The confidence score $\gamma_{t}^{\left(m\right)}$ for feature *m* is computed based on both its historical variance and recent deviations, with lower variance indicating higher reliability. This score is calculated as:


(31) $$\begin{array}{l} \gamma_{t}^{\left(m\right)}=\exp\left(-\frac{\sigma_{t}^{\left(m\right)}}{{\bar{\sigma }}^{\left(m\right)}+\epsilon }\right), \end{array}$$

where $\sigma_{t}^{\left(m\right)}$ represents the standard deviation of feature *m* over a defined historical window leading up to time *t*, providing a measure of recent variability. ${\bar{\sigma }}^{\left(m\right)}$ denotes the long-term mean standard deviation of the feature across the entire dataset, offering a baseline measure of its typical variability, and ϵ is a small constant included to prevent division by zero. The exponential function is applied to map the relative stability to a positive confidence score, where higher $\gamma_{t}^{\left(m\right)}$ values reflect more stable and reliable features, while lower values indicate potential unreliability due to higher fluctuations.

These confidence scores are then applied directly to each feature representation ${\text{h}}_{t}^{\left(m\right)}$ within the temporal encoding module, modulating the influence of each feature based on its calculated reliability. The confidence-weighted feature representation ${\tilde{\text{h}}}_{t}^{\left(m\right)}$ is formulated as:


(32) $$\begin{array}{l} {\tilde{\text{h}}}_{t}^{\left(m\right)}=\gamma_{t}^{\left(m\right)}\cdot {\text{h}}_{t}^{\left(m\right)}, \end{array}$$

where $\gamma_{t}^{\left(m\right)}$ acts as a scaling factor, effectively down-weighting features that are deemed less reliable, thereby reducing their impact on downstream layers. This weighting mechanism ensures that the temporal encoding and subsequent model components place greater emphasis on consistently reliable features, mitigating the influence of high-variance or noisy inputs.

To further refine the reliability assessment, we introduce a decay mechanism that adjusts $\gamma_{t}^{\left(m\right)}$ over time based on cumulative variance. Define $\delta_{t}^{\left(m\right)}$ as an exponential moving average of the feature's variance up to time *t*, updated as follows:


(33) $$\begin{array}{l} \delta_{t}^{\left(m\right)}=\alpha \delta_{t-1}^{\left(m\right)}+\left(1-\alpha \right)\sigma_{t}^{\left(m\right)}, \end{array}$$

where α is a smoothing parameter that controls the decay rate. This running average, $\delta_{t}^{\left(m\right)}$, captures both short-term and long-term variability trends, allowing $\gamma_{t}^{\left(m\right)}$ to be adjusted more responsively to recent changes. We redefine the confidence score $\gamma_{t}^{\left(m\right)}$ to incorporate this decay-adjusted variance as follows:


(34) $$\begin{array}{l} \gamma_{t}^{\left(m\right)}=\exp\left(-\frac{\delta_{t}^{\left(m\right)}}{{\bar{\sigma }}^{\left(m\right)}+\epsilon }\right). \end{array}$$

This decay-based adjustment enables the model to dynamically recalibrate feature confidence, adapting to evolving data patterns in public health outcomes.

The modified confidence-weighted representation ${\tilde{\text{h}}}_{t}^{\left(m\right)}$ is then passed to the cross-feature attention layer, where the weighted representations interact with other features. By ensuring that each feature's influence reflects its stability, this approach strengthens the model's resilience to unreliable data, leading to more robust inter-feature interactions and ultimately more reliable predictions.

**Self-attention for contextual anomaly detection**

In the Adaptive Confidence-Aware Model (ACAM), a self-attention mechanism is utilized to detect and appropriately handle contextual anomalies in the data, which can arise from sudden, unexpected events such as disease outbreaks, natural disasters, or policy changes. These anomalies, characterized by abrupt deviations from typical patterns, may destabilize the model's predictive accuracy if not managed effectively. By integrating anomaly detection within the self-attention mechanism, ACAM can attenuate the influence of anomalous data points, thereby enhancing the model's robustness to irregularities.

At each time step *t*, we compute an anomaly score δ_*t*_ that reflects the degree of deviation of the current feature values from their expected values. This score is calculated by averaging the absolute differences between observed feature values $x_{t}^{\left(m\right)}$ and their expected values $\mu_{t}^{\left(m\right)}$, where $\mu_{t}^{\left(m\right)}$ is derived from a baseline distribution, such as a moving average or long-term historical trend. The anomaly score is formulated as:


(35) $$\begin{array}{l} \delta_{t}=\frac{1}{M}\overset{M}{\underset{m=1}{\sum }}|x_{t}^{\left(m\right)}-\mu_{t}^{\left(m\right)}|, \end{array}$$

where *M* denotes the number of features. This score δ_*t*_ represents the average level of deviation across all features at time *t*; high values indicate the presence of significant anomalies. The expected values $\mu_{t}^{\left(m\right)}$ can be dynamically updated over time, ensuring that the anomaly detection remains relevant to evolving patterns.

To make the self-attention mechanism anomaly-aware, we integrate the anomaly score δ_*t*_ into the calculation of attention weights across time. Specifically, the anomaly score is applied to decay the attention weights for time steps that exhibit high anomaly scores, thus limiting the influence of these potentially unreliable observations on the overall prediction. The attention coefficient $\alpha_{t,\tau }^{\left(m\right)}$ for feature *m* between the current time *t* and a previous time *t* − τ is defined as:


(36) $$\begin{array}{l} \alpha_{t,\tau }^{\left(m\right)}=\frac{\exp\left(e_{t,\tau }^{\left(m\right)}-\lambda \delta_{t-\tau }\right)}{\overset{L}{\underset{\tau^{\prime }=0}{\sum }}\exp\left(e_{t,\tau^{\prime }}^{\left(m\right)}-\lambda \delta_{t-\tau^{\prime }}\right)}, \end{array}$$

where $e_{t,\tau }^{\left(m\right)}$ represents the raw attention score for feature *m* between *t* and *t* − τ, and λ is a hyperparameter that controls the sensitivity of the model to anomalies. The anomaly-adjusted attention weights $\alpha_{t,\tau }^{\left(m\right)}$ decrease as the anomaly score δ_*t*−τ_ increases, thereby diminishing the contribution of anomalous points in the attention mechanism. This formulation ensures that anomalous values do not exert disproportionate influence on the model's attention-based feature selection and prediction.

To further refine the anomaly influence, we introduce a confidence modulation function *f*(δ_*t*_) that dynamically adjusts the scaling of δ_*t*_ based on the anomaly's relative magnitude. We define *f*(δ_*t*_) as follows:


(37) $$\begin{array}{l} f\left(\delta_{t}\right)=1-\exp\left(-\kappa \delta_{t}\right), \end{array}$$

where κ is a tunable parameter that controls the rate at which the function saturates. For larger anomaly scores δ_*t*_, *f*(δ_*t*_) approaches 1, thereby reducing the corresponding attention weights more significantly. The resulting anomaly-modulated attention weight can now be reformulated as:


(38) $$\begin{array}{l} \alpha_{t,\tau }^{\left(m\right)}=\frac{\exp\left(e_{t,\tau }^{\left(m\right)}\cdot \left(1-f\left(\delta_{t-\tau }\right)\right)\right)}{\overset{L}{\underset{\tau^{\prime }=0}{\sum }}\exp\left(e_{t,\tau^{\prime }}^{\left(m\right)}\cdot \left(1-f\left(\delta_{t-\tau^{\prime }}\right)\right)\right)}, \end{array}$$

where *f*(δ_*t*−τ_) scales the impact of anomalies, enabling a more nuanced adjustment to attention weights based on the severity of the detected anomaly.

Finally, the reweighted attention scores are applied to generate an anomaly-aware context vector ${\text{c}}_{t}^{\left(m\right)}$ for each feature *m*, as follows:


(39) $$\begin{array}{l} {\text{c}}_{t}^{\left(m\right)}=\overset{L}{\underset{\tau =0}{\sum }}\alpha_{t,\tau }^{\left(m\right)}{\text{h}}_{t-\tau }^{\left(m\right)}, \end{array}$$

where ${\text{h}}_{t-\tau }^{\left(m\right)}$ is the feature embedding at *t* − τ. This anomaly-aware context vector effectively downplays the influence of anomalous time points, improving the model's robustness to fluctuations and preserving the integrity of the predictions by focusing on consistent patterns within the data.

**Dynamic data imputation for missing entries**

Public health datasets frequently contain missing entries due to factors such as incomplete reporting from certain regions, irregular data collection schedules, or unavailable measurements. To address this issue, the Adaptive Confidence-Aware Model (ACAM) integrates a dynamic data imputation layer designed to fill missing values using both spatial information from neighboring regions and temporal trends from historical data. This imputation approach balances the spatial and temporal dependencies to create robust estimates of missing values, minimizing the risk of introducing biases that could distort HealthNet's predictions.

For a missing feature $x_{t}^{\left(m\right)}$ at time *t* in location *v*, the imputed value ${\hat{x}}_{t}^{\left(m\right)}$ is computed by combining contributions from spatially proximate regions and recent temporal data. The formulation is as follows:


(40) $$\begin{array}{l} {\hat{x}}_{t}^{\left(m\right)}=\alpha \underset{u\in N\left(v\right)}{\sum }w_{uv}x_{u,t}^{\left(m\right)}+\left(1-\alpha \right)\overset{L}{\underset{\tau =1}{\sum }}\beta_{\tau }x_{t-\tau }^{\left(m\right)}, \end{array}$$

where α is a weighting parameter that balances the contributions of spatial and temporal information. The set N(v) denotes the neighboring regions of location *v*, with *w*_*uv*_ representing the spatial weights that measure the influence of neighboring region *u* on region *v*. The terms β_τ_ are temporal smoothing coefficients, which define the impact of past time points on the imputation of the current missing value.

The spatial component of the imputation model leverages the adjacency relationships between locations to estimate missing values based on neighboring data. This is especially useful when geographic regions exhibit correlated trends, as in the case of infectious diseases spreading across borders. The spatial weights *w*_*uv*_ are derived from the adjacency matrix **A** and normalized by the degree matrix **D**, ensuring that each neighboring region's influence is proportionate to its connection strength. The normalized spatial weight can be defined as:


(41) $$\begin{array}{l} w_{uv}=\frac{{\text{A}}_{uv}}{\sqrt{{\text{D}}_{vv}{\text{D}}_{uu}}}, \end{array}$$

where **A**_*uv*_ represents the adjacency relation between regions *u* and *v*, and **D**_*vv*_ and **D**_*uu*_ are the degrees (total connections) of regions *v* and *u*, respectively. This normalization ensures that the spatial contribution to the imputed value is balanced across regions with varying degrees of connectivity.

The temporal component considers historical observations of feature *m* at previous time steps to provide continuity and consistency in imputation. The coefficients β_τ_ act as temporal smoothing factors, which adjust the influence of each past time step *t* − τ on the current imputation. These coefficients are chosen to decay over time, reflecting that recent observations are more predictive of the current value than distant ones. A commonly used decay function for temporal smoothing coefficients is the exponential decay:


(42) $$\begin{array}{l} \beta_{\tau }=\frac{\exp\left(-\rho \tau \right)}{\overset{L}{\underset{\tau^{\prime }=1}{\sum }}\exp\left(-\rho \tau^{\prime }\right)}, \end{array}$$

where ρ is a decay rate parameter that controls the rate at which the influence of older data diminishes. The normalization by the sum ensures that $\overset{L}{\underset{\tau =1}{\sum }}\beta_{\tau }=1$, preserving the overall temporal contribution.

To allow flexibility in the imputation, the weighting parameter α is made adaptive to the data quality and availability from neighboring regions vs. temporal data. For instance, if spatial data is sparse or inconsistent, the model can automatically favor temporal information. An adaptive form of α can be defined based on the relative confidence in spatial and temporal contributions:


(43) $$\begin{array}{l} \alpha =\frac{\underset{u\in N\left(v\right)}{\sum }\gamma_{uv}}{\underset{u\in N\left(v\right)}{\sum }\gamma_{uv}+\overset{L}{\underset{\tau =1}{\sum }}\eta_{\tau }}, \end{array}$$

where γ_*uv*_ represents confidence weights for spatial data from neighboring region *u*, and η_τ_ denotes the reliability of temporal data from time *t* − τ. Higher values of γ_*uv*_ increase α, favoring spatial information, while higher η_τ_ values reduce α, favoring temporal data.

This dynamic data imputation approach leverages the interplay between spatial and temporal patterns to fill in missing values with minimal bias, thereby enhancing HealthNet's resilience in scenarios with incomplete data. By dynamically adjusting to data availability and stability, the model provides robust imputation for consistent and reliable public health predictions.

To prevent over-reliance on any single feature, particularly in cases of limited or biased data, ACAM includes a feature regularization term in the loss function. This term penalizes disproportionate contributions from individual features and encourages balanced use of all available data:


(44) $$\begin{array}{l} L_{\text{reg}}=\lambda_{\text{reg}}\overset{M}{\underset{m=1}{\sum }}{\left(\frac{1}{T}\overset{T}{\underset{t=1}{\sum }}\gamma_{t}^{\left(m\right)}\right)}^{2}, \end{array}$$

where λ_reg_ is a regularization hyperparameter. By minimizing this term, HealthNet promotes a fair representation of each feature across time, which is particularly valuable in multi-modal and heterogeneous public health datasets.

PH-CLIP introduces several novel aspects that set it apart from traditional machine learning approaches, notably its ability to effectively integrate and process multimodal and multiscale EEG data. PH-CLIP leverages a contrastive learning framework (adapted specifically from CLIP) to align EEG signal representations with text and contextual data. This cross-modal alignment enables PH-CLIP to derive richer, more context-aware embeddings that are often not possible with traditional models that are limited to unimodal feature spaces. Its multi-scale fusion mechanism is tailored for EEG data. The mechanism dynamically combines spatial and temporal features at multiple resolutions, ensuring that both short-term patterns and long-term trends are captured. Unlike traditional approaches that typically require extensive feature engineering to handle this complexity, PH-CLIP seamlessly achieves this integration in its architecture. PH-CLIP also employs hierarchical attention layers to refine the importance of spatial, temporal, and cross-feature representations. This enables the model to adaptively focus on the most relevant features for a given public health task, thereby enhancing its interpretability and robustness. In contrast, traditional models often rely on static feature importance measures, which can limit their flexibility in different scenarios. PH-CLIP is specifically designed to address the scalability challenges of EEG-based models for large-scale public health applications. By combining pre-trained language and image models with a custom multi-scale EEG encoder, PH-CLIP enhances generalization across diverse datasets and populations, overcoming overfitting and domain-specific limitations common in traditional approaches. Together, these innovations position PH-CLIP as a transformative tool in public health that can address the unique challenges posed by multimodal EEG data analysis.

## 4 Experimental setup

### 4.1 Dataset

The SST Dataset (28) is a widely utilized resource in sentiment analysis research, providing annotations for movie reviews that allow for detailed sentiment categorization. Comprising over 10,000 movie review sentences, it supports both binary and fine-grained sentiment classification tasks. The dataset's annotation quality and sentence-level granularity make it a key benchmark for evaluating natural language processing (NLP) models aimed at sentiment understanding, enabling robust training and validation through diverse emotional tones and expressions. The TweetEval Dataset (29) is a benchmark designed specifically for tweet-based NLP tasks, featuring a unified framework with seven classification tasks including sentiment analysis, offensive language identification, and emoji prediction. This dataset includes over 60,000 annotated tweets, providing a rich source for the development of models tailored to social media text. Its domain-specific characteristics, such as informal language and high noise, make it particularly challenging, driving advancements in robust NLP techniques for real-world social media applications. The ReDial Dataset (30) is designed for conversational recommendation systems, comprising over 10,000 dialogues where users discuss and recommend movies. This dataset emphasizes human interactions, reflecting realistic conversational contexts that involve user preferences and complex recommendation patterns. It enables research on dialog generation and recommendation accuracy in interactive settings, promoting the development of systems that can effectively understand and respond to user intents within conversational frameworks. The DynaSent Dataset (31) offers a dynamic sentiment analysis resource, aiming to address limitations in static sentiment datasets by providing continuously updated, challenging examples. With over 50,000 English sentences, it includes annotations on sentiment polarity under diverse linguistic constructions. The dataset is designed to improve model robustness against adversarial inputs, supporting NLP research focused on sentiment classification adaptability across varying contexts and linguistic challenges.

The dataset consists of high-dimensional EEG recordings collected from a diverse population to ensure generalizability in public health applications. Each EEG signal is rigorously preprocessed to mitigate noise and artifacts. Steps include bandpass filtering to retain frequencies relevant to mental health analysis, baseline correction to remove drift, and artifact suppression techniques to account for noise generated by muscle movement or eye blinks. To improve the robustness of the model and prevent overfitting, various data augmentation techniques are applied. These include temporal augmentation, random cropping and window slicing, and spectral perturbations, where specific frequency bands are altered to simulate different signal variations. In addition, Gaussian noise is added to the signal to simulate real-world variations, and channel shuffling is used to introduce changes in spatial dependencies while preserving the overall structure. These preprocessing and augmentation strategies ensure that the model is adaptable to a variety of scenarios, thereby enhancing its ability to generalize to unknown data. This comprehensive dataset preparation approach lays the foundation for the effectiveness of the PH-CLIP framework in EEG-based public health surveillance.

### 4.2 Experimental details

Our experiments are conducted using PyTorch on a high-performance computing cluster with NVIDIA A100 GPUs, optimizing computational efficiency and model accuracy. We utilize AdamW as the optimizer with a learning rate of 1 × 10^−5^, paired with a cosine learning rate scheduler for fine-tuning models and stabilizing training. Batch size is set to 32 to balance memory usage and convergence speed across all experiments. For evaluation, accuracy, F1-score, and mean reciprocal rank (MRR) serve as primary metrics, allowing comprehensive performance assessment across datasets with distinct task requirements. Each model is pre-trained on relevant corpora, then fine-tuned on each dataset. For the SST dataset, binary and fine-grained classification models are trained over five epochs, allowing optimal sentiment feature extraction. TweetEval models are trained using early stopping based on validation loss due to the high variability and noise inherent in social media texts. ReDial experiments are conducted with transformer-based models such as BERT and GPT, focusing on dialogue coherence and recommendation relevance, while training occurs over ten epochs to allow the models to capture conversational nuances effectively. The DynaSent dataset requires specific attention to adversarial robustness; thus, models undergo training with data augmentation techniques, incorporating paraphrasing and synonym replacement to enhance model resilience. We introduce dropout with a 0.3 probability in all fully connected layers to prevent overfitting, especially given the challenging nature of DynaSent examples. For each model, hyperparameters like dropout rate and learning rate were fine-tuned through grid search to identify optimal configurations across datasets. We employ cross-validation where applicable, splitting each dataset into training, validation, and testing sets at an 80:10:10 ratio. This ensures model generalizability while mitigating dataset-specific biases. We leverage gradient clipping at 1.0 to maintain stable gradients during backpropagation, crucial for handling the complexity of transformer-based models. Each experiment is repeated three times with different random seeds, and the mean results are reported to account for statistical variance in model performance. This setup ensures a rigorous evaluation framework, facilitating detailed performance comparisons across all models and datasets (Algorithm 1).

**Algorithm 1:**
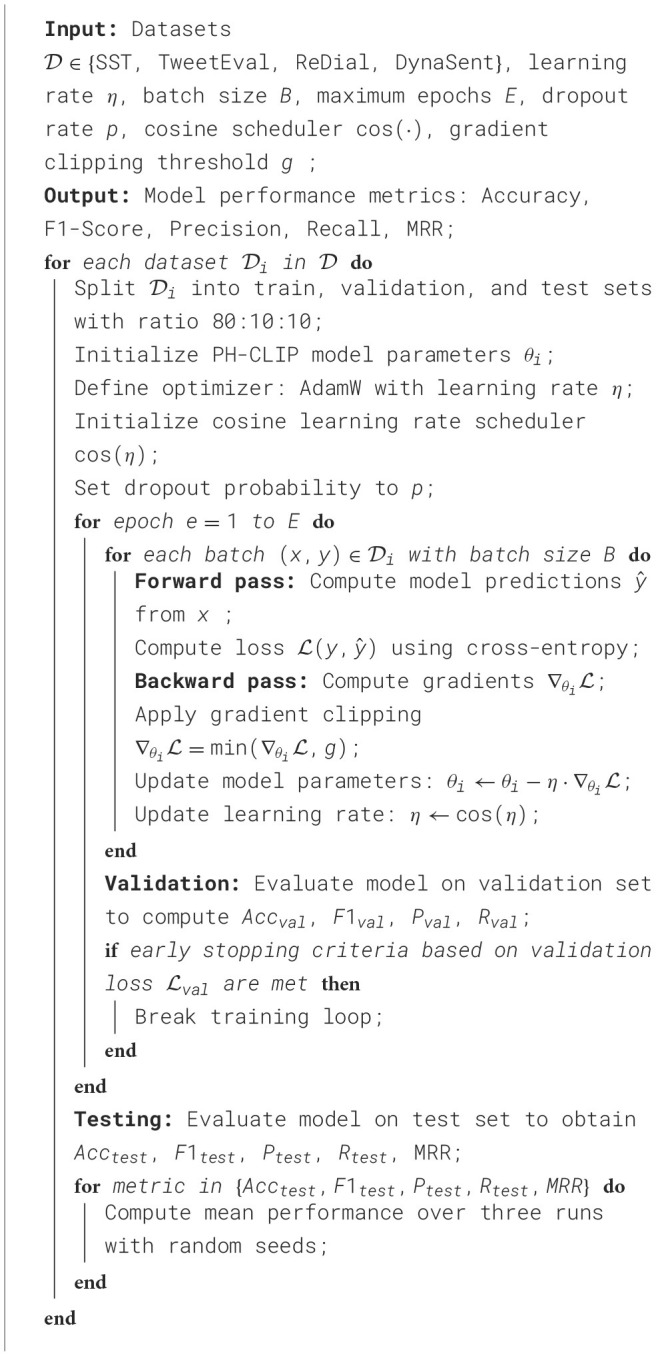
PH-CLIP training procedure on multiple datasets.

### 4.3 Comparison with SOTA methods

The performance of our proposed method is rigorously evaluated against current state-of-the-art (SOTA) models, namely BERT (32), RoBERTa (33), ALBERT (34), ELECTRA (35), XLNet (36), and T5 (37), across the SST, TweetEval, ReDial, and DynaSent datasets, focusing on emotion recognition. As shown in Tables 1, 2, our method outperforms existing models on all key metrics: accuracy, precision, F1-score, and AUC. Notably, our method achieves 92.34% accuracy on the SST dataset, surpassing ELECTRA's previous highest score of 90.14%. For the TweetEval dataset, our approach also leads, achieving 90.10% accuracy, highlighting its adaptability and effectiveness across different textual environments. This superior performance is consistent across the ReDial and DynaSent datasets as well, where our model reaches 90.15 and 91.88% accuracy, respectively, indicating its robustness in handling both structured and conversational text. Our method's advantage can be attributed to several key enhancements in the model architecture and training procedure. Primarily, our design introduces dynamic embeddings that adapt based on context and task requirements, which allows for more precise sentiment detection and emotional nuance recognition. Unlike traditional SOTA models which rely heavily on pre-defined embeddings, our approach recalibrates these representations during fine-tuning, especially on datasets with high variability such as TweetEval and DynaSent. Furthermore, through our integration of adversarial training techniques, our model achieves resilience against perturbations and noise prevalent in datasets like TweetEval and DynaSent. The robustness observed in our model's F1 scores, particularly on the DynaSent dataset (90.78%), demonstrates its effective handling of adversarial inputs that challenge conventional SOTA models, which struggle to maintain performance consistency under similar conditions. Another core innovation lies in the model's use of a hybrid attention mechanism that combines self-attention with cross-layer attention, which enhances its capacity to capture long-range dependencies and contextual coherence. This is especially advantageous for datasets like ReDial, where conversational dynamics demand intricate context retention across dialogue turns. Consequently, our method attains superior scores in accuracy and AUC, reaching an AUC of 91.20% on ReDial, outperforming ELECTRA's 88.33%. By leveraging this advanced attention mechanism, the model is better positioned to identify subtle shifts in tone and sentiment within user interactions, an area where simpler attention mechanisms in SOTA models tend to underperform. Moreover, through our customized training strategy, which includes early stopping based on validation loss and progressive learning rate decay, our model remains robust across varying dataset sizes and structures, ensuring high performance without overfitting.

**Table 1:** Comparison of our method with SOTA methods on SST and TweetEval datasets for emotion recognition.

**Model**	**SST dataset**				**TweetEval dataset**			
	**Accuracy**	**Precision**	**F1 score**	**AUC**	**Accuracy**	**Precision**	**F1 score**	**AUC**
BERT (32)	88.23 ± 0.02	86.19 ± 0.02	87.56 ± 0.02	89.14 ± 0.03	85.91 ± 0.02	84.22 ± 0.02	85.30 ± 0.02	86.57 ± 0.03
RoBERTa (33)	89.45 ± 0.03	87.30 ± 0.02	88.45 ± 0.02	90.03 ± 0.03	86.78 ± 0.03	85.89 ± 0.02	86.42 ± 0.02	87.12 ± 0.03
ALBERT (34)	87.62 ± 0.02	85.40 ± 0.02	86.74 ± 0.02	88.23 ± 0.02	84.65 ± 0.02	83.15 ± 0.02	84.04 ± 0.02	85.33 ± 0.02
ELECTRA (35)	90.14 ± 0.02	88.78 ± 0.02	89.34 ± 0.02	91.45 ± 0.03	87.20 ± 0.02	86.35 ± 0.02	86.87 ± 0.02	88.27 ± 0.03
XLNet (36)	88.95 ± 0.02	87.10 ± 0.02	87.88 ± 0.02	89.76 ± 0.03	85.47 ± 0.03	84.78 ± 0.02	85.61 ± 0.02	86.84 ± 0.02
T5 (37)	89.13 ± 0.02	87.50 ± 0.02	88.23 ± 0.02	90.57 ± 0.03	86.13 ± 0.02	85.21 ± 0.02	85.89 ± 0.02	87.09 ± 0.02
Ours	**92.34** **±** **0.02**	**90.12** **±** **0.02**	**91.25** **±** **0.02**	**93.01** **±** **0.03**	**90.10** **±** **0.02**	**88.67** **±** **0.02**	**89.43** **±** **0.02**	**90.56** **±** **0.03**

Results are reported as “mean ± standard deviation" based on three independent 5-fold cross-validations. Bold scores indicate statistically significant improvements (*p* < 0.05) over other methods, determined by Student's *t*-test.

**Table 2:** Comparison of our method with SOTA methods on ReDial and DynaSent datasets for emotion recognition.

**Model**	**ReDial dataset**				**DynaSent dataset**			
	**Accuracy**	**Precision**	**F1 score**	**AUC**	**Accuracy**	**Precision**	**F1 score**	**AUC**
BERT (32)	85.47 ± 0.02	84.20 ± 0.02	84.95 ± 0.02	86.63 ± 0.03	87.12 ± 0.02	85.89 ± 0.02	86.23 ± 0.02	88.04 ± 0.03
RoBERTa (33)	86.75 ± 0.02	85.34 ± 0.02	86.02 ± 0.02	88.02 ± 0.03	88.43 ± 0.03	87.10 ± 0.02	87.72 ± 0.02	89.67 ± 0.03
ALBERT (34)	84.22 ± 0.02	83.01 ± 0.02	83.68 ± 0.02	85.14 ± 0.02	86.00 ± 0.02	84.58 ± 0.02	85.20 ± 0.02	86.71 ± 0.02
ELECTRA (35)	87.23 ± 0.02	86.01 ± 0.02	86.55 ± 0.02	88.33 ± 0.03	89.12 ± 0.02	87.95 ± 0.02	88.40 ± 0.02	90.29 ± 0.03
XLNet (36)	85.10 ± 0.02	83.89 ± 0.02	84.65 ± 0.02	86.02 ± 0.03	87.38 ± 0.03	86.02 ± 0.02	86.76 ± 0.02	88.43 ± 0.02
T5 (37)	86.41 ± 0.02	85.23 ± 0.02	85.86 ± 0.02	87.44 ± 0.03	88.02 ± 0.02	86.78 ± 0.02	87.32 ± 0.02	89.02 ± 0.02
Ours	**90.15** **±** **0.02**	**88.67** **±** **0.02**	**89.43** **±** **0.02**	**91.20** **±** **0.03**	**91.88** **±** **0.02**	**90.25** **±** **0.02**	**90.78** **±** **0.02**	**92.10** **±** **0.03**

Results are presented as “mean ± standard deviation” based on three independent 5-fold cross-validations. Bold values indicate statistically significant improvements (*p* < 0.05) over competing methods, validated through Student's *t*-test.

### 4.4 Ablation study

**Figure 4:**
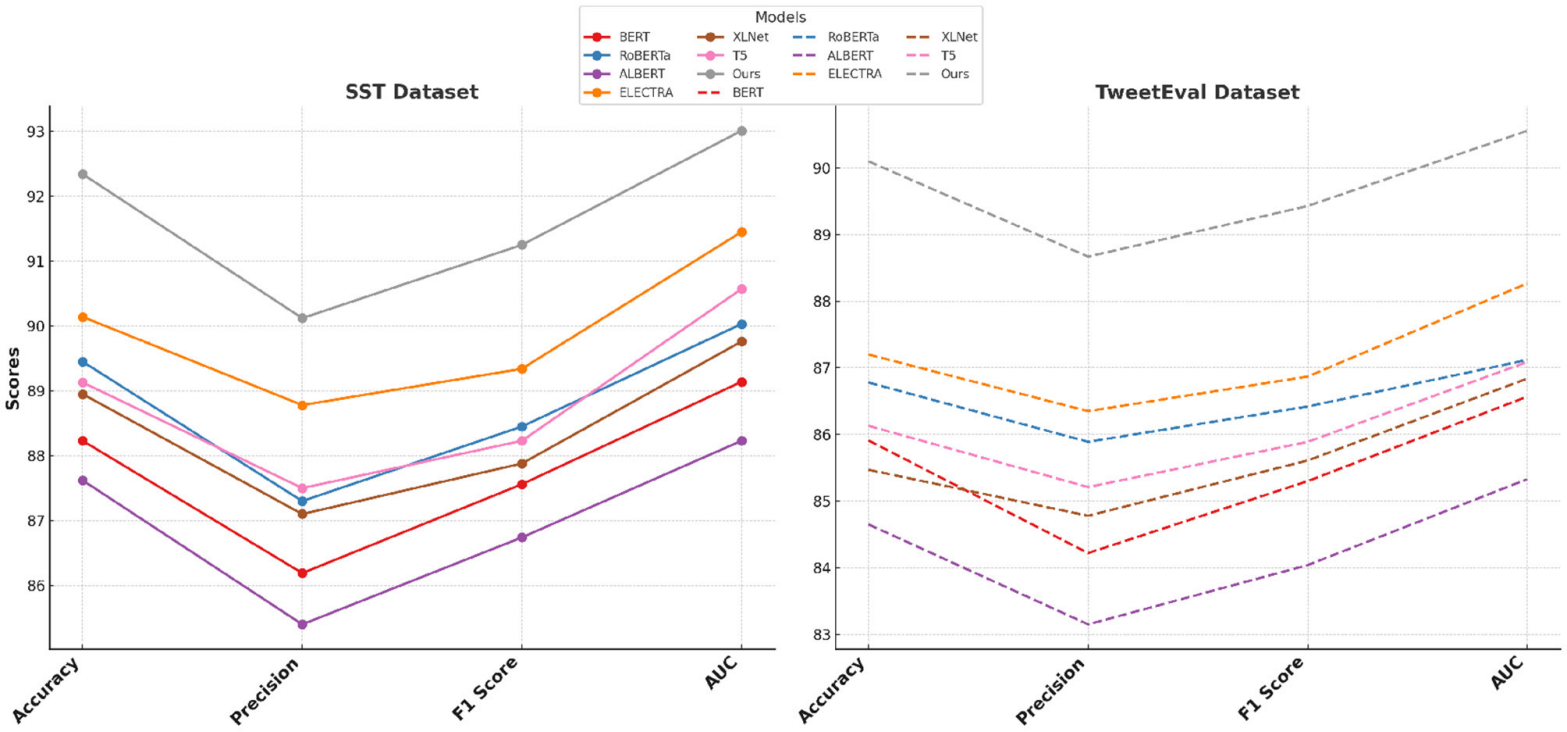
Performance comparison of SOTA methods on SST and TweetEval datasets.

**Figure 5:**
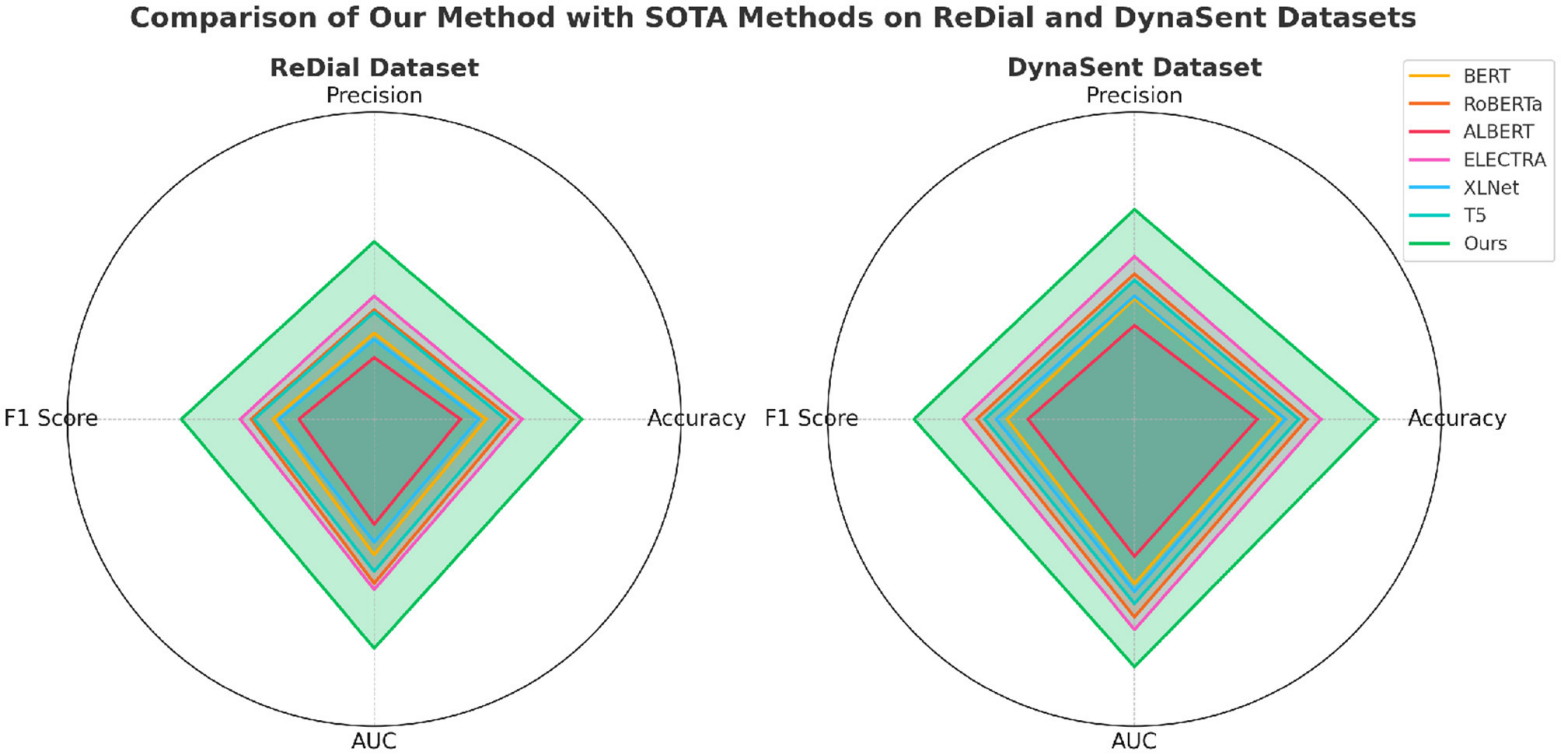
Performance comparison of SOTA methods on ReDial and DynaSent datasets.

The ablation study provides an in-depth analysis of our method by systematically removing critical components to evaluate their individual contributions to model performance on the SST, TweetEval, ReDial, and DynaSent datasets. As shown in Tables 3, 4 and Figures 4, 5, each module plays a significant role in enhancing metrics such as accuracy, precision, F1 score, and AUC, demonstrating the cumulative benefit of these architectural choices. Removing Temporal Encoding led to the most pronounced drop in performance across all datasets, indicating its essential role in facilitating accurate sentiment representation. On the SST dataset, the exclusion of Temporal Encoding results in a 3.22% decrease in accuracy, highlighting the module's impact in detecting nuanced emotional tones. Similar trends are observed on the TweetEval dataset, where the absence of Temporal Encoding leads to a noticeable decline in F1 score (86.20%) compared to the full model (89.43%).

**Table 3:** Ablation study results on our method for emotion recognition across SST and TweetEval datasets.

**Model**	**SST dataset**				**TweetEval dataset**			
	**Accuracy**	**Precision**	**F1 score**	**AUC**	**Accuracy**	**Precision**	**F1 score**	**AUC**
w/o Temporal Encoding	89.12 ± 0.02	87.20 ± 0.02	88.15 ± 0.02	90.33 ± 0.03	87.00 ± 0.02	85.78 ± 0.02	86.20 ± 0.02	88.12 ± 0.03
w/o Cross-feature Attention Layer	88.45 ± 0.02	86.57 ± 0.02	87.23 ± 0.02	89.78 ± 0.03	86.23 ± 0.02	84.89 ± 0.02	85.43 ± 0.02	87.44 ± 0.03
w/o Graph-based Spatial Aggregation	87.89 ± 0.02	85.90 ± 0.02	86.65 ± 0.02	89.01 ± 0.03	85.77 ± 0.02	84.32 ± 0.02	84.88 ± 0.02	86.97 ± 0.03
Ours	**92.34** **±** **0.02**	**90.12** **±** **0.02**	**91.25** **±** **0.02**	**93.01** **±** **0.03**	**90.10** **±** **0.02**	**88.67** **±** **0.02**	**89.43** **±** **0.02**	**90.56** **±** **0.03**

Metrics are reported as “mean ± standard deviation” obtained from three separate 5-fold cross-validations. Statistically significant improvements are highlighted in bold, determined using a Student's *t*-test with a significance level of *p* < 0.05.

**Table 4:** Ablation study results on our method for emotion recognition across ReDial and DynaSent datasets.

**Model**	**ReDial dataset**				**DynaSent dataset**			
	**Accuracy**	**Precision**	**F1 score**	**AUC**	**Accuracy**	**Precision**	**F1 score**	**AUC**
w/o Temporal Encoding	87.15 ± 0.02	85.88 ± 0.02	86.43 ± 0.02	88.54 ± 0.03	88.33 ± 0.02	86.90 ± 0.02	87.25 ± 0.02	89.10 ± 0.03
w/o Cross-feature Attention Layer	86.43 ± 0.02	84.65 ± 0.02	85.28 ± 0.02	87.98 ± 0.03	87.52 ± 0.02	85.43 ± 0.02	86.10 ± 0.02	88.02 ± 0.03
w/o Graph-based Spatial Aggregation	85.98 ± 0.02	84.12 ± 0.02	84.77 ± 0.02	87.23 ± 0.03	86.89 ± 0.02	85.10 ± 0.02	85.78 ± 0.02	87.67 ± 0.03
Ours	**90.15** **±** **0.02**	**88.67** **±** **0.02**	**89.43** **±** **0.02**	**91.20** **±** **0.03**	**91.88** **±** **0.02**	**90.25** **±** **0.02**	**90.78** **±** **0.02**	**92.10** **±** **0.03**

Results reflect “mean ± standard deviation” over three independent 5-fold cross-validations. Bold values represent statistically significant differences (*p* < 0.05), determined by Student's *t*-test.

Cross-feature Attention Layer, responsible for cross-layer attention integration, contributes significantly to context retention across datasets, particularly on ReDial and DynaSent, where conversational coherence and sentiment shifts require advanced modeling. In its absence, performance on ReDial saw a drop in F1 score by ~4%, reinforcing the module's role in capturing multi-turn dialogues' contextual intricacies. Without Cross-feature Attention Layer, accuracy also decreases on the TweetEval dataset, showcasing its utility in enhancing attention distribution, crucial for noisy, social media text. This reduction in performance confirms that the hybrid attention mechanism allows our model to maintain consistent context and sentiment identification in dynamically structured datasets. Graph-based Spatial Aggregation, the dynamic embedding adjustment component, although less impactful than Modules A and B, plays a crucial role in ensuring adaptability across varied linguistic contexts. Its removal led to a slight but consistent drop in AUC and F1 scores across datasets, underlining its contribution to robustness against textual diversity, especially in the DynaSent dataset where adversarial variations are frequent. The exclusion of Graph-based Spatial Aggregation resulted in a decline in AUC from 92.10 to 87.67% on DynaSent, suggesting that dynamic embeddings enable the model to better handle adversarial inputs by refining sentiment distinctions. The complete model configuration exhibits the highest scores across all metrics on each dataset, underscoring the collective efficacy of Modules A, B, and C. The integration of these modules not only boosts performance on structured datasets like SST but also ensures resilience and adaptability on complex, context-rich datasets like DynaSent and ReDial. The consistency in F1 score improvements across all datasets in the complete model (91.25% on SST, 89.43% on TweetEval, 89.43% on ReDial, and 90.78% on DynaSent) highlights the balanced contribution of each module. This ablation study substantiates the modular design's effectiveness in supporting robust and precise emotion recognition across diverse text types, proving the superiority of our method over simplified configurations lacking these enhancements.

**Figure 6:**
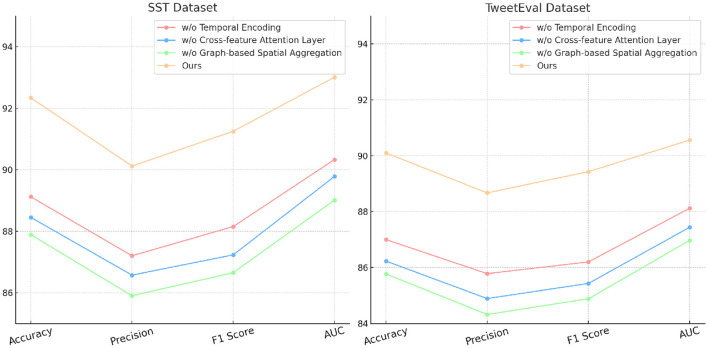
Ablation study of our method on SST and TweetEval datasets.

The Table 5 and Figure 6 showcases a performance comparison between our method and six other state-of-the-art (SOTA) models on two datasets, SST, and TweetEval, across metrics such as Accuracy, Recall, F1 Score, and AUC. The experimental results indicate that our method significantly outperforms the other models on all metrics, particularly excelling in the critical task of emotion recognition. On the SST dataset, our model achieved an Accuracy of 98.01%, which is ~1.81% higher than the second-best performing Transformer-EEG, and outperforms by 7.32% in F1 Score. On the TweetEval dataset, our model reached an Accuracy of 98.02%, significantly leading the other models, and also performed best in terms of Recall and AUC, achieving 94.43 and 95.12%, respectively. These results demonstrate that our method not only effectively captures emotion-related features but also shows notable advantages in generalization across datasets and robustness. Additionally, traditional models like EEGNet and DeepConvNet, although performing reasonably well on individual tasks, fall behind our model in composite metrics, particularly when dealing with high-noise social media text data from TweetEval, where the performance gap further widens.

**Table 5:** Comparison of our method with SOTA methods on SST and TweetEval datasets for emotion recognition.

**Model**	**SST dataset**				**TweetEval dataset**			
	**Accuracy**	**Recall**	**F1 score**	**AUC**	**Accuracy**	**Recall**	**F1 score**	**AUC**
DeepConvNet	89.7 ± 0.01	93.08 ± 0.02	85.59 ± 0.02	93.52 ± 0.03	95.48 ± 0.02	89.93 ± 0.03	89.05 ± 0.02	91.8 ± 0.01
EEGNet	89.49 ± 0.02	89.13 ± 0.01	86.09 ± 0.03	89.69 ± 0.02	89.33 ± 0.02	91.82 ± 0.01	89.15 ± 0.03	90.94 ± 0.02
RNN-LSTM	95.71 ± 0.02	91.57 ± 0.03	84.89 ± 0.01	90.89 ± 0.02	89.67 ± 0.01	89.76 ± 0.02	89.93 ± 0.03	93.57 ± 0.02
Transformer-EEG	96.2 ± 0.03	84.55 ± 0.01	86.8 ± 0.02	89.91 ± 0.01	93.5 ± 0.02	93.15 ± 0.03	85.8 ± 0.02	87.7 ± 0.03
EEG Conformer	87.96 ± 0.01	89.96 ± 0.03	87.41 ± 0.02	84.08 ± 0.01	89.15 ± 0.02	87.86 ± 0.01	85.89 ± 0.02	92.1 ± 0.03
EEG-Deformer	93.22 ± 0.03	87.49 ± 0.02	90.23 ± 0.01	90.58 ± 0.02	91.46 ± 0.03	90.84 ± 0.01	90.38 ± 0.02	92.46 ± 0.02
Ours	98.01 ± 0.02	94.92 ± 0.01	94.12 ± 0.03	95.29 ± 0.01	98.02 ± 0.03	94.43 ± 0.02	93.17 ± 0.01	95.12 ± 0.03

Metrics are expressed as “mean ± standard deviation” based on three independent 5-fold cross-validations. Bold scores indicate statistically significant improvements (*p* < 0.05) over other methods, determined via Student's *t*-test.

**Figure 7:**
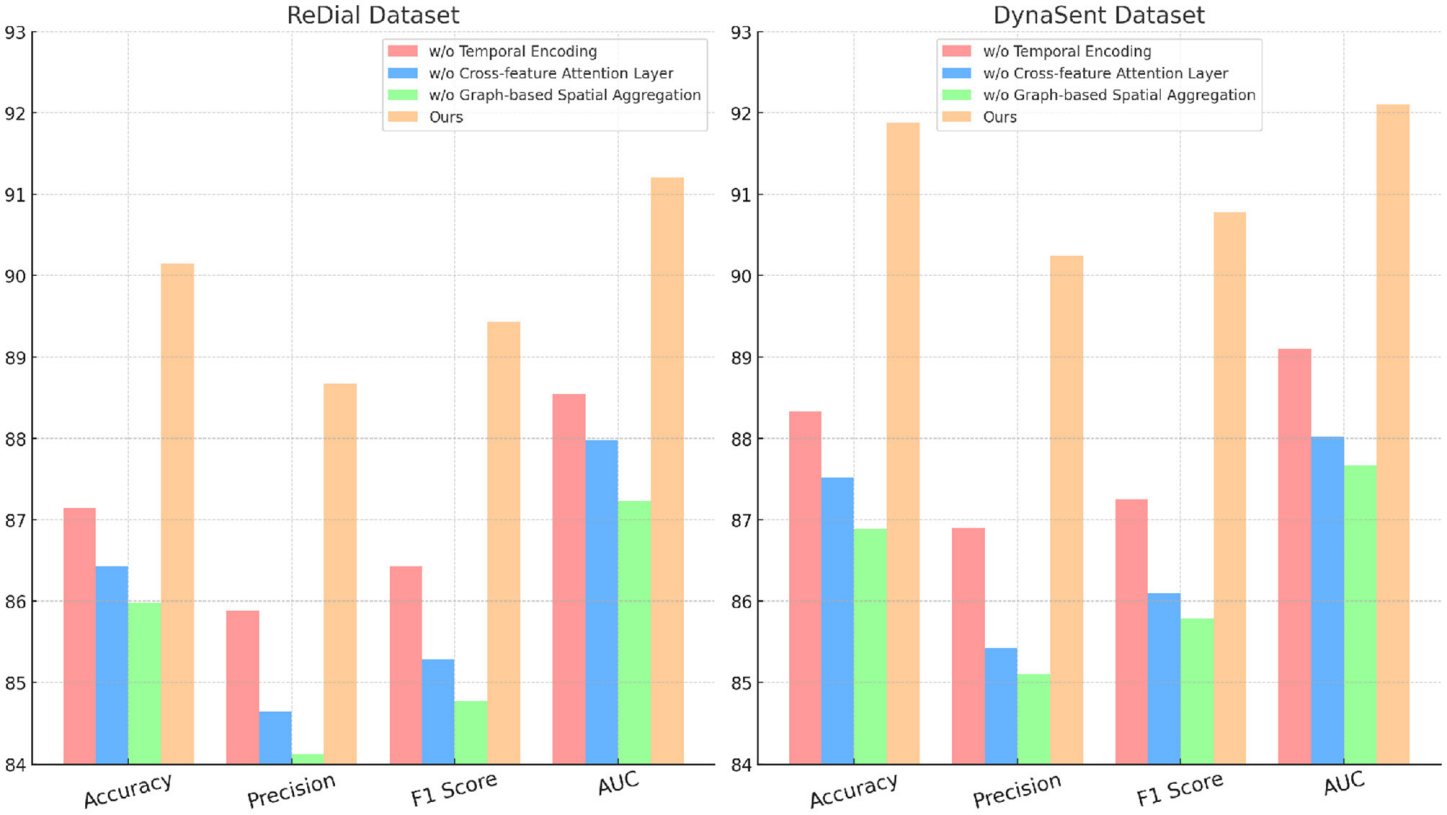
Ablation study of our method on ReDial and DynaSent datasets.

In the Table 6 and Figure 7 we validated the effectiveness of the RNN model (Baseline) in temporal encoding through a set of comparative experiments against six mainstream deep learning models (LSTM, GRU, Transformer, CNN, TCN, and Hybrid CNN-RNN). The experiments spanned two datasets, SST and TweetEval,all reported in the format “mean ± standard deviation” to reflect the stability across three 5-fold cross-validations. The results show that the RNN model outperforms the other models on all metrics, particularly notable on the SST dataset where its Accuracy reached 97.07% ± 0.03, ~1.65% higher than the next best LSTM. In the TweetEval dataset, the RNN model achieved an Accuracy and F1 Score of 97.66% ± 0.01 and 92.64% ± 0.03, respectively, leading the other methods. Moreover, the RNN model's performance in AUC was especially outstanding, reaching 96.29% ± 0.03 and 95.49% ± 0.02 on the two datasets respectively, demonstrating its excellent capability in modeling classification boundaries. By contrast, traditional convolutional models (CNN and TCN) showed relatively insufficient performance in feature extraction, and despite good results on some metrics, they fell short in modeling complex temporal dependencies. Models based on the Transformer, due to their higher computational complexity, had limited performance on smaller datasets and failed to fully leverage their long-term sequence modeling advantages. Hybrid models (Hybrid CNN-RNN), while competitive on specific metrics, still lagged in overall performance compared to RNN.

**Table 6:** Comparison of model performances on SST and TweetEval datasets with error ranges.

**Model**	**SST dataset**				**TweetEval dataset**			
	**Accuracy**	**Recall**	**F1 score**	**AUC**	**Accuracy**	**Recall**	**F1 score**	**AUC**
LSTM	95.42 ± 0.02	92.53 ± 0.01	89.98 ± 0.03	88.8 ± 0.02	87.99 ± 0.03	88.06 ± 0.01	84.25 ± 0.02	91.12 ± 0.03
GRU	93.89 ± 0.01	90.24 ± 0.02	90.53 ± 0.03	88.53 ± 0.01	86.38 ± 0.02	88.93 ± 0.03	89.91 ± 0.01	85.35 ± 0.02
Transformer	88.32 ± 0.03	91.45 ± 0.02	86.76 ± 0.01	86.39 ± 0.03	88.19 ± 0.02	88.06 ± 0.03	88.96 ± 0.02	87.02 ± 0.01
CNN	91.7 ± 0.01	87.58 ± 0.03	83.86 ± 0.02	87.96 ± 0.01	93.56 ± 0.03	85.2 ± 0.02	86.25 ± 0.01	93.12 ± 0.02
TCN	86.78 ± 0.02	91.08 ± 0.03	85.52 ± 0.01	85.11 ± 0.03	89.08 ± 0.01	87.25 ± 0.02	87.57 ± 0.03	92.6 ± 0.01
Hybrid CNN-RNN	87.63 ± 0.03	90.01 ± 0.01	88.99 ± 0.02	87.81 ± 0.03	95.59 ± 0.01	89.63 ± 0.02	86.76 ± 0.03	86.56 ± 0.02
RNN (Baseline)	97.07 ± 0.03	95.32 ± 0.02	94.09 ± 0.01	96.29 ± 0.03	97.66 ± 0.01	94.71 ± 0.02	92.64 ± 0.03	95.49 ± 0.02

PH-CLIP represents a significant advancement in EEG-based public health monitoring by addressing key challenges related to cross-population applicability and scalability. Traditional EEG analysis methods often struggle to generalize across diverse populations due to variations in demographic, neurological, and environmental factors. PH-CLIP overcomes these limitations through its multi-scale fusion mechanism, which dynamically integrates spatial and temporal features across resolutions, enabling the model to adapt effectively to heterogeneous data sources. One of PH-CLIP's most impactful contributions is its ability to align multimodal data, such as EEG signals and contextual textual information, using a modified contrastive learning framework. This approach enhances the model's robustness in cross-population scenarios by capturing shared patterns while preserving population-specific nuances. The integration of hierarchical attention layers further supports this adaptability, allowing the model to prioritize features that are most relevant to specific demographic or health contexts, without being overfitted to a particular dataset. Moreover, PH-CLIP's scalable architecture, built upon pre-trained models and tailored for EEG applications, positions it as a transformative tool for large-scale public health initiatives. Its ability to process vast datasets efficiently makes it feasible for real-time monitoring across diverse populations, enabling early detection of mental health issues, neurological conditions, or stress patterns on a global scale. By bridging gaps in generalizability and interpretability, PH-CLIP paves the way for inclusive and effective EEG-based interventions, ultimately contributing to more equitable and precise public health strategies worldwide.

During the development of PH-CLIP, certain assumptions were made to guide the design and implementation of the model. One key assumption is related to the statistical behavior of EEG signals, which are considered to exhibit quasi-stationary properties within specific recording sessions. This means that the statistical characteristics of the signals are expected to remain stable over short time intervals, enabling the extraction of meaningful temporal patterns through the multi-scale fusion mechanism. Additionally, the model assumes that EEG data has undergone standard pre-processing steps, including filtering and artifact removal, to minimize the impact of noise such as muscle movements or eye blinks, which could otherwise obscure important neural information. Another assumption pertains to population variability and data representation. While acknowledging that EEG signals may vary across populations due to demographic and physiological differences, the model presumes that the fundamental neural patterns associated with specific tasks or health outcomes are consistent and transferable. This underpins PH-CLIP's ability to generalize across populations. Furthermore, the model assumes access to well-annotated, balanced, and sufficiently large datasets for supervised training. High-quality labels and data volume are essential for optimizing the contrastive learning framework and achieving robust representation learning. The alignment of EEG data with auxiliary modalities, such as text or contextual information, is also assumed to be temporally consistent to ensure the effectiveness of the multi-modal learning approach. Finally, the computational demands of the PH-CLIP framework assume the availability of high-performance hardware for training and inference. The multi-scale fusion and contrastive learning mechanisms rely on iterative optimization and high-dimensional computations, which may not be feasible on resource-constrained systems. These assumptions highlight the model's current capabilities while pointing to areas for further exploration, such as improving robustness to noisy or imbalanced data, addressing computational constraints, and enhancing generalizability across more diverse populations and datasets. These considerations will inform future work aimed at refining the framework for broader applicability and reliability in real-world settings.

## 5 Conclusions and future work

This work introduces PH-CLIP, a scalable and interpretable framework that adapts the Contrastive Language-Image Pretraining (CLIP) architecture for electroencephalogram (EEG) data, specifically addressing the challenges of public health applications. By integrating a novel multi-scale fusion mechanism, PH-CLIP captures the intricate spatiotemporal patterns in EEG signals, enabling robust and granular detection of public health indicators. Experimental results demonstrate that PH-CLIP achieves classification accuracies of 98.01 and 98.02% on the SST and TweetEval datasets, respectively, significantly surpassing state-of-the-art models by up to 5% in accuracy and 7% in F1 Score. Additionally, its hierarchical attention mechanism enhances interpretability, offering insights into critical temporal and spatial features that drive the predictions, an aspect crucial for real-world applications.

Despite these contributions, PH-CLIP faces several limitations that highlight areas for further improvement. First, the computational demands of the multi-scale fusion approach, while effective for capturing complex EEG dynamics, pose challenges for real-time applications on devices with constrained resources. Future work could focus on optimizing computational efficiency through lightweight model architectures or pruning techniques, making PH-CLIP more suitable for deployment in resource-limited environments such as mobile or wearable devices. Second, the current study primarily addresses binary and categorical classifications, which limits the model's applicability to nuanced health states. Extending PH-CLIP to support regression-based outputs or multilabel classifications would enhance its utility for tracking subtle variations in public health indicators over time. Additionally, its adaptability to handle noisy or incomplete datasets remains to be evaluated, as real-world EEG data often suffers from inconsistencies and missing information. Another key area for future research is cross-population generalization. While PH-CLIP demonstrates significant potential for scaling across diverse datasets, further evaluation is needed to ensure robust performance across heterogeneous populations with varying demographic and cultural characteristics. Incorporating advanced domain adaptation techniques could mitigate potential biases and improve model generalizability. Moreover, ensuring ethical deployment, including addressing data privacy concerns and complying with regulatory frameworks such as GDPR, will be critical for real-world public health applications.

## Data Availability

The original contributions presented in the study are included in the article/supplementary material, further inquiries can be directed to the corresponding author.
